# A Functional Variant at a Prostate Cancer Predisposition Locus at 8q24 Is Associated with *PVT1* Expression

**DOI:** 10.1371/journal.pgen.1002165

**Published:** 2011-07-21

**Authors:** Kerstin B. Meyer, Ana-Teresa Maia, Martin O'Reilly, Maya Ghoussaini, Radhika Prathalingam, Patricia Porter-Gill, Stefan Ambs, Ludmila Prokunina-Olsson, Jason Carroll, Bruce A. J. Ponder

**Affiliations:** 1 Cancer Research UK Cambridge Research Institute, Cambridge, United Kingdom; 2 Department of Oncology, University of Cambridge, Cambridge, United Kingdom; 3 Laboratory of Translational Genomics, Division of Cancer Epidemiology and Genetics, National Cancer Institute, National Institutes of Health, Bethesda, Maryland, United States of America; 4 Laboratory of Human Carcinogenesis, Center for Cancer Research, National Cancer Institute, National Institutes of Health, Bethesda, Maryland, United States of America; Fred Hutchinson Cancer Research Center, United States of America

## Abstract

Genetic mapping studies have identified multiple cancer susceptibility regions at chromosome 8q24, upstream of the *MYC* oncogene. *MYC* has been widely presumed as the regulated target gene, but definitive evidence functionally linking these cancer regions with *MYC* has been difficult to obtain. Here we examined candidate functional variants of a haplotype block at 8q24 encompassing the two independent risk alleles for prostate and breast cancer, rs620861 and rs13281615. We used the mapping of DNase I hypersensitive sites as a tool to prioritise regions for further functional analysis. This approach identified rs378854, which is in complete linkage disequilibrium (LD) with rs620861, as a novel functional prostate cancer-specific genetic variant. We demonstrate that the risk allele (G) of rs378854 reduces binding of the transcription factor YY1 *in vitro*. This factor is known to repress global transcription in prostate cancer and is a candidate tumour suppressor. Additional experiments showed that the YY1 binding site is occupied *in vivo* in prostate cancer, but not breast cancer cells, consistent with the observed cancer-specific effects of this single nucleotide polymorphism (SNP). Using chromatin conformation capture (3C) experiments, we found that the region surrounding rs378854 interacts with the *MYC* and *PVT1* promoters. Moreover, expression of the *PVT1* oncogene in normal prostate tissue increased with the presence of the risk allele of rs378854, while expression of *MYC* was not affected. In conclusion, we identified a new functional prostate cancer risk variant at the 8q24 locus, rs378854 allele G, that reduces binding of the YY1 protein and is associated with increased expression of *PVT1* located 0.5 Mb downstream.

## Author Summary

Genome-wide association studies have been very successful at identifying gene loci that are associated with common diseases. However for many of these it has been very difficult to elucidate the identity and function of the risk variant and how altered function predisposes to disease. A 1.2-Mb gene-poor region upstream of the *MYC* oncogene has been shown to contain at least 12 risk loci associated with different types of cancer. In this study, we use the analysis of chromatin conformation as a tool to identify sub-regions within this 1.2 Mb region that are involved in the control of gene expression, and we identify molecular mechanisms which may confer risk of different specific cancer types. In particular, we examine a risk locus that is associated with predisposition to prostate cancer and identify a DNA sequence variation that results in a change in the binding site for the nuclear factor YY1 as a potential causative mechanism. We show that the risk locus is able to interact with two downstream genes, *MYC* and *PVT1*, and present evidence that *PVT1* is a candidate new target gene regulated by the 8q24 risk region.

## Introduction

Genome-wide association studies (GWAS) have identified multiple independent cancer susceptibility loci upstream of the *MYC* oncogene at chromosome 8q24 (reviewed in [1]): initial studies revealed one breast [2], three prostate [3], one bladder cancer [4] locus and one region conferring risk for multiple cancers including prostate, colon and ovarian cancer. More recently, a lymphocytic leukaemia [5] and five more prostate risk regions have been identified [3], [6], [7]. The 8q24 region is currently considered the most important susceptibility region for prostate cancer, accounting for about 8% of the two-fold increase in risk observed in first degree relatives [3]. *MYC* ranks as one of the most consistently overexpressed genes comparing prostate tumours to normal prostate tissue in a meta-analysis of five data sets [8], indicating its importance in prostate cancer. Breast tumours and metastatic prostate tumours carry frequent amplifications of the 8q24 region, spanning a large region covering both the *MYC* and the neighbouring *PVT1* gene [9], [10]. Furthermore, both the *MYC* and the *PVT1* genes are frequent targets for retroviral integration in mouse tumour assays [11]. The *MYC* oncogene functions as a transcriptional activator, and is part of a complex regulatory network controlling cell growth, apoptosis, differentiation and other cellular responses [12]. *PVT1* encodes a non-coding RNA and is a host gene for several miRNAs, namely hsa-miR-1204, 1205, 1206 and 1207 [11], [13]. The targets and function of *PVT1* and its embedded miRNAs are still largely unknown.

The study presented here focuses on a haplotype block containing a risk variant for prostate cancer (rs620861) [3] and a second independent risk variant for breast cancer (rs13281615) [2]. Other cancer risk loci at 8q24 have already been examined in some detail. For example, the colorectal and prostate cancer predisposition locus contains an enhancer that is able to interact with the *MYC* gene and it was suggested that rs6983267 within this enhancer alters a TCF7L2 (TCF4) binding site thus resulting in changed sensitivity to WNT signalling [14], [15]. Transgenic assays indicate that rs6983267 increases expression of a reporter gene in a pattern that mimics *MYC* activity in the mouse prostate [16]. Another prostate cancer-associated variant from the 8q24 region, rs11986220, has been shown to form a FoxA1 site, leading to an increased cancer risk because of stronger androgen responsiveness [17]. However, when examining gene expression in primary human prostate and colon tissue samples, no correlation between *MYC* expression and either of these SNPs was found [14], [15], [18], [19], but an allele-specific effect of rs6983267 was detectable in some colorectal cancer cell lines [20].

The majority of cancer susceptibility loci identified by GWAS do not affect coding regions of genes, and are thought to be regulatory. However, the identification of functional SNPs has been difficult since tagging SNPs employed in genetic mapping are in tight LD with many SNPs, often covering large haplotype blocks. In this study, we hypothesized that regulatory elements affected by SNPs are likely to be positioned in regions of active chromatin that are accessible for digestion by DNase I [21]. Thus, we mapped DNase I hypersensitive sites (DHSs) as means of prioritising regions for further functional analysis. Previously, this approach successfully showed that the likely causative SNPs in both the *FGFR2* and *TNRC9* susceptibility loci are in regions of open chromatin [22], [23]. Here, we find that rs378854, which is in perfect LD with the prostate cancer risk SNP rs620861, maps to a highly accessible site. We show that the cancer risk-associated allele of rs378854 decreases binding of the transcription factor YY1, activates *in vitro* expression of reporter constructs relative to the non-risk allele, and increases expression of *PVT1* in primary normal human prostate tissue. Therefore, in addition to *MYC*, our study implicates *PVT1*, located almost 0.5 Mb from the functional variant, as a novel candidate cancer gene whose expression is influenced by 8q24 genetic variation.

## Results

**Figure 1:**
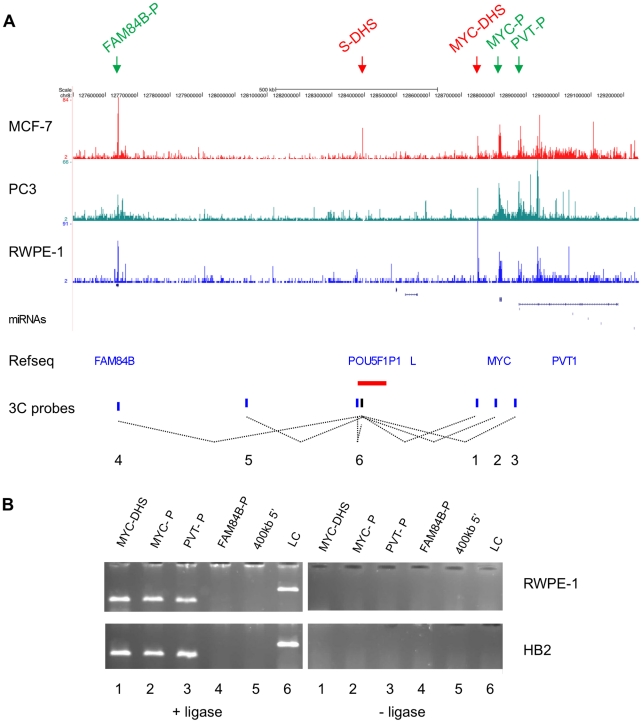
Chromatin conformation at the 8q24 locus. (A) DNase-seq track for the breast cancer cell line MCF-7, the prostate cancer cell line PC3 and the normal-like prostate cell line RWPE-1 displayed on the UCSC genome browser for a 1.7 MB region (chr8: 127,550,000-129,250,000) surrounding the 8q24 cancer susceptibility region, where peaks indicate regions of increased sensitivity to digestions with DNase I. Reads were adjusted for the total number of reads in each experiment. DHSs and promoters used in subsequent analysis are marked by arrows. RefSeq genes are shown, with L being LOC727677. The prostate and breast cancer susceptibility region is depicted by a horizontal red bar. Dotted lines show interactions tested in the chromatin conformation capture experiment shown in (B). (B) Chromatin conformation capture (3C) experiment using the S-DHS as bait in the HB2 breast and the RWPE-1 prostate cell lines. Target sequences are indicated above the lanes. A target sequence just upstream of the *Eco*RI site 5′ to the bait *Eco*RI site was used as ligation control (LC). Negative controls for each ligation reaction were cut but unligated DNA fragments. The numbers below the panel refer to interactions shown in (A).

The mapping of DHSs using DNase-seq in the breast cancer cell line MCF-7 across the entire 8q24 cancer susceptibility region revealed a prominent site of DNase I sensitivity within the haplotype block that carries a breast and a prostate cancer predisposition variant, rs13281615 and rs620861, respectively (Figure 1A, Figure S1). The strength of the signal for the DHS within this susceptibility block (S-DHS) is similar to that seen for promoter regions of the *FAM84B*, *MYC* and *PVT1* genes (Figure 1A). In addition, a strong DHS was observed in a conserved region 60kb upstream of the *MYC* gene (MYC-DHS). The DNase-seq results were compared to results obtained in DNase-chip experiments (Figure S2). All strong DHSs, for example at the *MYC* promoter, were replicated. However, the S-DHS which maps to a repetitive element could not be detected by DNase-chip, as repetitive sequences are excluded from the array by design. Interestingly, the same DNase-seq approach applied to prostate cancer cell lines RWPE-1 and PC3 (Figure 1A) did not detect a signal at the S-DHS location, suggesting that transcription factor occupancy in this region may differ between breast and prostate cancer cell lines. Signals at other locations, for example at the *MYC* promoter are comparable in all three cell lines (Figure 1A). Figure S3A shows an enlarged view of the region, confirming that the S-DHS is in an open chromatin state in MCF-7 cells, but in a closed state in two prostate cell lines. Despite being located within a LINE element, the sequence of S-DHS is unique enough to specifically align it to this genomic locus (see Figure S3 and S4 for details). To confirm that the open chromatin at the S-DHS is indicative of the binding of regulatory nuclear proteins, we examined chromatin immunoprecipitation data (ChIP-seq) for MCF-7 cells [24]. Figure S5A and S5B show that S-DHS sequences are bound by the two cohesin subunits Rad21 and SA1, by CTCF and the transcription factor FoxA1, making it highly likely that this region acts as a regulatory element in MCF-7 cells. Sequence alignments for ChIP-seq, as for the DNase-seq, are sufficiently specific to uniquely map the signals to this region.

Next, we analysed the 1.2 kb S-DHS region for the presence of genetic variants that could be linked to variants previously reported to be associated with breast or prostate cancer. The 1000 Genomes database included 12 SNPs, of which only 5 were common (MAF >0.05) (Figure S2). Of these, rs378854 showed complete LD (r^2^ = 1) with rs620861, the most strongly associated prostate cancer SNP reported by Al Olama *et al.* [3] (independently confirmed by Yeager et al. [6]), and also with rs445114, reported in Gudmundsson et al. [7], while the other variants displayed only low LD with this SNP. There was no strong connection to breast cancer in this region, as rs378854 only ranks as number 23 of all SNPs tested in this haplotype block [25]. Previous work and LD analysis of this region (Figure S6) supports the presence of two independent functional variants within this haplotype block, one for prostate cancer and one for breast cancer [3], [8]. Thus, we conclude that the closed state of chromatin within the S-DHS might be associated with risk of prostate cancer, through rs378854.

**Figure 2:**
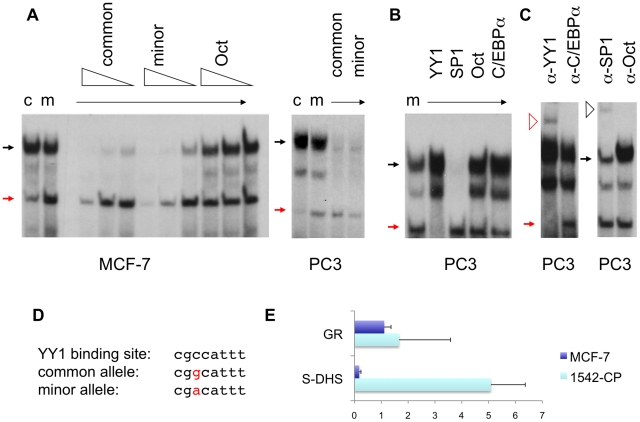
Protein DNA interactions at the sequences overlapping rs378854. (A) Electrophoretic mobility shift assays (EMSA) were carried out with oligonucleotides containing the common (c) and minor (m) alleles of rs378854 and nuclear extracts from the breast cancer cell line MCF-7 and the prostate cancer cell line PC3. Competitor oligonucleotides were present at 100-, 30- and 10-fold excess for MCF-7 and 30-fold excess for PC3 and are indicated above the lanes. The red and black closed arrows denote the YY1 and SP1 complexes, respectively. (B) EMSAs of the oligonucleotide containing the minor allele were carried out with PC3 nuclear extract and 30-fold excess of competitor oligonucleotides as shown. (C) Supershift of the complex using polyclonal antibody against YY1, SP1, Oct-1 and C/EBPα with PC3 nuclear extracts. The red and black open arrows denote the YY1 and SP1 supershift complexes. (D) An alignment of the two alleles with a YY1 binding site [28] is shown. (E) Chromatin immunoprecipitation assay showing the fold-enrichment of the S-DHS and the positive control (glucocorticoid receptor, GR) sequences relative to a negative control (S-DHS-ve) after immunoprecipitation with YY1 antibody in MCF-7 breast 1542-CP prostate cancer cells.

Motif prediction algorithms suggest that the minor, non-risk allele (A) of rs378854 creates either a YY1 or a C/EBPα transcription factor binding site. Using electrophoretic mobility shift assays (EMSA) with nuclear extracts from breast and prostate cancer cells (MCF-7 and PC3) we demonstrate that the oligonucleotide probe overlapping rs378854 is able to interact with several nuclear proteins (Figure 2A). The low mobility band (black arrow) is not affected by the SNP. In contrast, the high mobility band (red arrow) binds the minor allele more strongly in both cell types. A third, more variable complex of intermediate mobility is formed, which is not affected by the presence of the SNP. The specificity of binding was confirmed by competition assays with self, non-self and unrelated Oct-1 probe at different concentrations (Figure 2A). Competition with known transcription factor binding sites suggest that the high mobility band contains the transcription factor YY1 (Figure 2B). This is confirmed by a supershift observed after including a YY1 antibody in the reaction (open red arrow, Figure 2C). The two upper complexes are sensitive to excess SP1 probe (Figure 2B), but only one of these complexes was supershifted by an SP1 antibody (black open arrow, Figure 2C). SP1 and YY1 are known to interact physically and function co-operatively to modify chromatin structure [26]. The SP1 binding site may therefore be able to enhance the allelic differences caused by YY1 binding to SNP rs378854. Using chromatin immunoprecipitation (ChIP) we also confirm that the identified YY1 site is occupied *in vivo* in the prostate cancer cell line 1542-CP, but not in MCF-7 breast cancer cells (Figure 2E). In 1542-CP cells YY1 occupancy at rs378854 is higher than that observed for a positive control fragment from the promoter region of the glucocorticoid receptor, known to contain three independent YY1 binding sites [27]. To test for allele-specific interaction between YY1 and rs378854 in 1542-CP, we attempted an allele-specific ChIP. Although there was an increase of binding towards the A allele in the majority of experiments, the effect was small and technical limitations prevent us from drawing definitive conclusions from this experiment.

**Figure 3:**
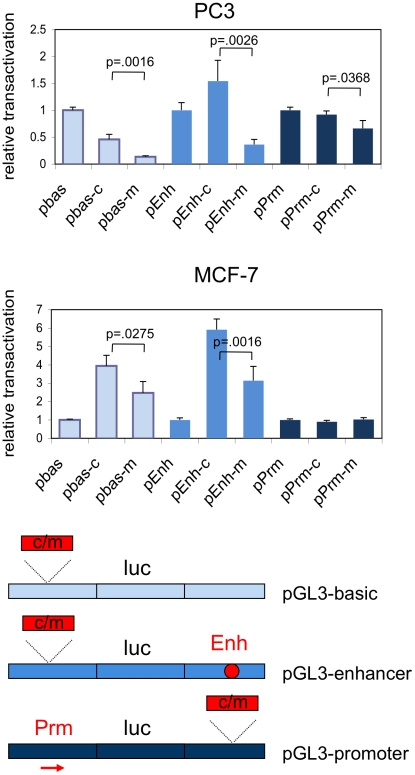
Relative transcriptional activation by the common and minor alleles of rs378854. The 395 bp fragment overlapping the S-DHS was cloned into pGL3-basic (pbas), pGL3-enhancer (pEnh) and pGL3-promoter (pPrm) and transcriptional activation of the reporter luciferase gene by the two alleles (common and minor) assayed in the PC3 prostate cancer and the MCF-7 breast cancer cell lines. Activation by the insert is given relative to the parental vector. Averages of six independent transfections are shown and the standard deviation for each data set is given in the bar chart. Where significant, p-values are given for a Student's t-test comparing the values obtained with common versus the minor allele in each vector background.

The S-DHS was identified in two breast cancer cell lines, T47 and MCF-7 (Figure S7). To examine the function of the S-DHS, we cloned a 395 bp fragment central to the S-DHS encompassing either the common (risk) or the minor (non-risk) allele of rs378854, into the pGL3-basic, pGL3-promoter and pGL3-enhancer vectors and assayed the ability of these allelic constructs to influence transcription in transient reporter assays. In the prostate cancer cell line PC3 the fragment containing the common (risk) allele has moderate ability to activate transcription in the context of the pGL3-enhancer construct, but for the minor allele there was statistically significant evidence for repression of transcription in the context of all constructs assayed (Figure 3). Our observation are consistent with previous reports that YY1 can act as a potent repressor of transcription [28]. In the breast cancer cell line MCF-7, the minor (protective) allele again displayed lower transcriptional activation than the common allele, but relative to the parental construct no repression was observed (Figure 2E). When the S-DHS was assayed in the pGL3-promoter vector the presence of the minor versus the common SNP had no effect in MCF-7 cells. Thus, rs378854 showed cell-type specific allelic effects on regulation of expression. The effect was evident in prostate cancer PC3 cells, with significant repressor activity of the protective minor allele. This may explain why rs378854 is associated with prostate, but not breast cancer susceptibility.

The S-DHS maps to a 1.2 MB region with very few annotated genes (Figure 1A). However, experiments for several 8q24 predisposition regions have indicated that this region is capable of undergoing long-range chromatin looping [14], [29]. We therefore used chromatin conformation capture (3C) to examine whether the DHS can physically interact with its neighbouring genes, *MYC*, *PVT1* and *FAM84B*. The pseudogene *POU5F1P1* and *LOC727677* are not expressed in prostate cells at detectable levels [17] and were therefore not included in this analysis. These experiments showed that in both the normal-like breast cell line HB2 and in the prostate cell line RWPE-1 the region surrounding the S-DHS interacts with a DHS 60 kb upstream of the *MYC* gene (MYC-DHS) and with the *MYC* and *PVT1* promoters, located 360 kb, 420 kb and 480 kb 3′ of the bait sequence, respectively (Figure 1). Similar results were obtained in MCF-7 cells (data not shown). There was no interaction with either the *FAM84B* promoter or with a negative control sequence 400 kb 5′ of the DHS (Figure 1B). Our results suggest that in prostate cells both *MYC* and *PVT1* could be target genes of the S-DHS regulator element. We note that both cohesin and CTCF also bind to the *MYC* and *PVT1* promoters in MCF-7 cells [24], suggesting a mechanism by which an interaction may occur.

**Figure 4:**
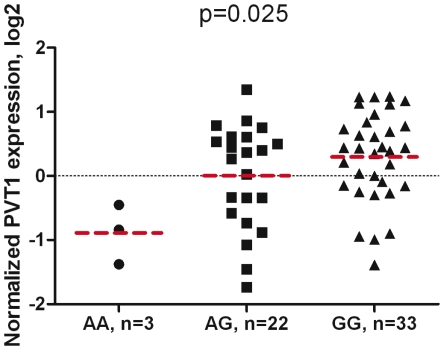
Association of *PVT1* gene expression with rs378854 genotype in 59 normal prostate samples. P-values are for univariate linear regression analysis of *PVT1* expression in relation to 0, 1 and 2 risk allele (G) of rs378854, adjusted for race. Expression values are shown on log2 scale relative to a mean value of all samples. Expression is lowest in carriers of non-risk AA genotypes and highest in carriers of risk GG genotypes. Mean expression values of each group are shown as bars.

Expression of *MYC* has been analysed extensively in human primary prostate and colon samples, but no correlation between its expression and the genotype of six different risk SNPs was identified [18], [19]. In contrast, our study revealed that in a set of 59 normal prostate samples mRNA expression level of *PVT1* increased with the presence of the risk allele at rs378854 (p = 0.025) (Figure 4). The trend was similar for individuals of European-American and African-American origin, even though the frequency of the risk G allele was 0.66 in Europeans and 0.90 in African-Americans (data not shown). As *PVT1* is located in a region of frequent genomic amplification, we examined copy-number variation (CNV) for intron 1 of *PVT1* on DNA from all tissue samples used for expression studies. *PVT1* CNV was not significantly associated with *PVT1* mRNA expression and the association for rs378854 was not affected by the CNV adjustment (Figure S8). At the same time, there was no evidence for association between *MYC* expression and rs378854 in these samples (p = 0.274). While this analysis will have to be repeated in larger sample sets, our results are consistent with a model in which disruption of YY1 binding at the common risk allele of rs378854 is associated with transcriptional activation of *PVT1*. We also examined expression of miRNAs embedded within the *PVT1* locus (hsa-mir-1204, hsa-mir-1205, hsa-mir-1206, hsa-mir-1207-3p and hsa-mir-1207-5p), but the expression was found to be very low or undetectable in both normal and tumour prostate samples and no clear pattern of association emerged (data not shown). However, we found hsa-mir-1208, located 50 kb downstream of *PVT1*, to be expressed in all prostate samples tested. In samples homozygous for the risk allele the expression was borderline increased in normal samples (n = 58, p = 0.042), while being decreased in tumour samples (n = 17, p = 0.068), demonstrating significant interaction effect for hsa-mir-1208 expression dependent on tissue status (normal/tumour) and rs378854 genotype (n = 75, p_int = 0.020, Figure S9). Similar analysis of hsa-mir-1208 expression in relation to previously reported rs6983267 did not reveal any significant associations in sets of normal and tumour samples, and in interaction (data not shown). The role of hsa-mir-1208 expression in prostate cancer and its long-distance regulation by rs378854 warrant further studies. In summary, we observe both a physical interaction between the risk SNP rs378854 and the *PVT1* promoter and an association between genotype and *PVT1* expression.

## Discussion

We describe here the identification of a novel transcriptional regulator element within the 8q24 region that is associated with expression of *PVT1*. The risk region is marked by a strong DHS in breast cancer MCF-7 cells and is able to interact physically with both the *MYC* and the *PVT1* promoter, but only *PVT1* and not *MYC* gene expression correlates with the presence of the risk allele. Within the S-DHS we identify rs378854 as a functional variant for prostate cancer susceptibility. We demonstrate that, when tested *in vitro*, using nuclear extracts from breast and prostate cancer cell lines, the YY1 protein was able to bind the non-risk allele (G) of 378854, a perfect proxy for the initially reported prostate cancer signal rs620861 [3], [6], more strongly than the risk allele. However, when tested *in vivo*, occupancy by YY1 was only observed in a prostate cancer cell line (1542-CP) but not in a breast cancer cell line (MCF-7) raising the possibility that the presence of YY1 prevents the establishment of a DHS. YY1, a zinc-finger transcription factor, is known to interact with chromatin remodelling enzymes which are thought to mediate some of YY1's functions [22]. Furthermore we observed that, in the context of the S-DHS, the protective allele of rs378854 was able to repress transcription in the prostate cell line PC3, but not in breast cancer MCF-7 cells. Therefore, we suggest that differential binding of YY1 to the prostate-cancer associated variant rs378854 might be functionally important for the regulation of *MYC* and/or *PVT1* expression. While *MYC* expression was not associated with rs378854, *PVT1* expression did correlate with the presence of the risk allele of rs378854 (p = 0.025). The absence of an association between *MYC* expression and the risk genotype may indicate that either the physical interaction between the risk region and the *MYC* promoter is functionally less important or secondary to the effect of *PVT1*, or that *MYC* expression is subject to additional control mechanisms, such as cell cycle or developmental regulation, which may mask the underlying association.

With respect to clinical prognosis, it is interesting to note that lower expression of nuclear YY1 correlates with a poorer outcome for prostate cancer [30], with the data suggesting that decreased YY1 levels give metastatic cells a survival advantage. YY1 has been reported to control many aspects of cancer biology through its interaction with cell cycle genes, association with p53 and other oncogenes as well as its regulation of key apoptosis-related molecules [28]. Furthermore, YY1 expression may play a role in both sensitivity and resistance to chemo- and immunotherapy [28].

**Figure 5:**
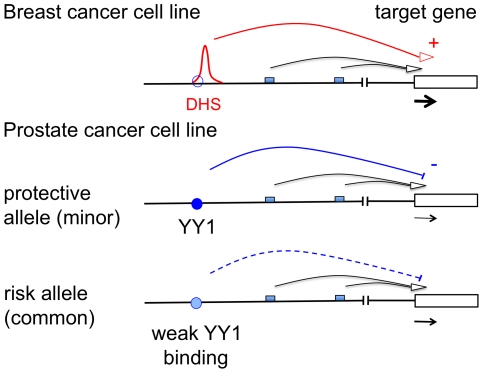
Schematic representation of the mode of action of rs378854. In a breast cancer cell line rs378854 is not bound by YY1 (site shown as empty blue circle) and the DHS present in this region is likely to act as a transcriptional regulator. In contrast, in a prostate cancer cell line YY1 binds (filled blue circle) and represses transcription of a target gene. The level of repression is influenced by the presence of the SNP in the YY1 binding site. Small boxes represent multiple other regulatory elements known to exist in the region.

Although risk intervals at 8q24 can affect the development of many different cancer types, it is striking that individual risk loci predispose to only a specific type of cancer. We observe that the enhancer identified within the colon/prostate/ovarian susceptibility region (rs696983267, shown in red in Figure S1) maps to a DHS in the prostate and colon, but not in the breast cancer cell lines analysed (Figure S10). However, the situation for the risk SNP we examine here is more complicated. We describe a DHS likely to mark a transcriptional enhancer in breast cancer cell lines, but suggest that a SNP lying within this element increases susceptibility to prostate cancer. We find that the YY1 site overlapping the risk SNP is occupied in a prostate but not a breast cancer cell line and propose that this binding of YY1 in prostate cells mediates transcriptional repression that is influenced by the presence of the SNP. Figure 5 depicts our model of action of rs378854 in the different cell lines. Interestingly, binding of YY1 does not appear to cause the formation of a DHS in the prostate cell lines. The available ENCODE data suggests that the majority of transcription factors bind to open chromatin. However, there is precedent for transcription factor binding to regions of closed chromatin. For example, approximately 40% of genome-wide FOXA1 binding sites map to regions of closed chromatin, where presumably the factor can exert changes in the chromatin structure in response to cell signalling or developmental cues [31]. For many transcription factors, including YY1, the correlation between site occupancy and chromatin accessibility has not yet been examined. Furthermore, the S-DHS is clearly subject to regulation since we detect it only in breast cancer cell lines. Histone modification data (UCSC genome browser) suggests that this region may also be accessible in human embryonic stem cells. In contrast, the majority of DHS elements are accessible across different tissue types. The regulatory elements in the 8q24 desert are clearly highly complex, tissue-specific and may be affected by the presence of SNPs. Here we have indentified one such variant, rs378854, but additional variants and other mechanisms, such as chromosome amplification and/or rare variants, may also contribute to cancer risk.

Previous experiments have implicated *MYC* as the target gene of the 8q24 susceptibility region [14]–[16], [18], [19]. However, in many primary tissue samples examined the presence of the risk allele does not affect *MYC* expression [18], [19]. Using allelic expression it has been possible to establish such a correlation, but only for some colon cancer cell lines [20]. Here we present evidence that one of the 8q24 risk loci for prostate cancer is able to interact with the promoter of *PVT1* and that the presence of the risk allele correlates with increased *PVT1* gene expression in normal prostate tissue. The *PVT1* gene encodes a non-coding transcript implicated in tumourigenesis [32], [33]. *PVT1* was identified as a locus co-amplified with *MYC* in human Burkitt's lymphomas and found to be a *MYC* activator [34]. In fact, we observed significant correlation between *MYC* and *PVT1* expression, r^2^ = 0.54 (p = 0.021 in 18 prostate tumours) and r^2^ = 0.34 (p = 0.0073 in 59 normal prostates). Therefore, the cancer-associated variants might eventually affect *MYC* expression, but indirectly, through *PVT1*. Furthermore, miRNAs encoded within *PVT1* may regulate *MYC* expression either directly or indirectly, by regulating factors that activate *MYC* [11]. However, the expression of these miRNAs appears to be low or undetectable in normal and tumour prostate tissue samples. On the other hand, a miRNA located 50 kb downstream of *PVT1*, hsa-mir-1208, showed an interesting pattern of expression and interaction between sample status (normal/tumour) and genotype of rs378854. This interesting association warrants further studies. Biochemical analysis of the *PVT1* promoter suggests that it is a target gene of *MYC* [35]. Importantly, silencing of *PVT1* transcripts activates apoptosis in cell lines with *PVT1* amplifications, while silencing of *MYC* in the same cell lines has no effect, suggesting that *PVT1* may play a direct role in tumourigenesis that is independent of *MYC* [10]. In this respect, it is interesting to note that *PVT1* shows increased expression in prostate cell lines compared to normal prostate tissue [17]. Furthermore, genetic variants within *PVT1* have been associated with Hodgkin's lymphoma [36].

It is striking that the S-DHS element potentially exerts its effect over large genomic distances, being located approximately 420 kb and 480 kb from the *MYC* and *PVT1* promoters, respectively. There are two large “gene deserts” upstream and downstream of the *MYC-PVT1* gene cluster (Figure S11), both containing extensive sequence conservation as well as multiple cancer predisposition loci, suggesting that these gene deserts are in fact highly complex regulatory regions of DNA, as supported by recent findings of multiple prostate enhancer elements within the *MYC* upstream region [17]. The identification of the repressor protein YY1 as a mediator of protection from cancer susceptibility is a step towards a better understanding of the function of this genomic region.

## Materials and Methods

### Ethics

Anonymised tumour and normal adjacent to tumour prostate tissue samples were either received from the National Cancer Institute (NCI) Cooperative Prostate Cancer Tissue Resource (CPCTR) or from the University of Maryland under protocols reviewed and approved by Institutional Review Boards.

### Cell culture

Breast cancer cell lines MCF-7, MDA-MB-134 (also referred to as MDA134), PMC42, T47D, normal-like breast cell line HB2, prostate cancer cell lines PC3 and 1542-CP, normal-like prostate cell line RWPE-1 and the HCT116 colon cancer cell line were from the Cambridge Research Institute culture collection. T47D, MDA134, PMC42, PC3 and HCT116 were maintained in RPMI, 10% foetal calf serum (FCS) and antibiotics; MCF-7 and HB2 in DMEM, 10% FCS and antibiotics, but HB2 cell medium was supplemented with insulin (5 µg/ml) and hydrocortisone (1 µg/ml) (Sigma, UK). RWPE-1 cells were cultured in Keratinocyte Serum Free Medium (Gibco) and supplemented with 5 ng/ml human recombinant EGF and 0.05 mg/ml bovine pituitary extract (both from Sigma). 1542-CP cells were cultured in keratinocyte serum-free medium (Keratinocyte-SFM, Life Technologies, Grand Island, NY) containing 25 µg/ml bovine pituitary extract, 5 ng/ml epidermal growth factor, 2 mM L-glutamine, 10 mM HEPES buffer, antibiotics, and 5% heat-inactivated FCS.

### DNase I hypersensitivity assay

Nuclei of all cell lines were harvested and digested with DNase I as previously described [22]. For the DNase-seq experiment DNA from DNase-digested nuclei was isolated by standard procedures, separated on a 1.4% agarose gel and gel-purified DNA fragments between 125 and 500 bp were used directly in Illumina pre-amplification. The amplified library was again resolved on an agarose gel to select 200–300 bp fragments that were gel-purified and sequenced using an Illumina Genome Analyser II, according to manufacturer's instructions. MCF-7 DNase-seq was carried out as part of our analysis of ER responses. Multiple MCF-7 aliquots were transfected with and without a siRNA to GATA3, but no differences in the DHS profile were detected. BWA software [37] was used for sequence alignment by the CRI Bioinformatics core facility. A representative experiment with low background is shown. Two independent DNase-seq experiments for PC3 and RWPE-1 gave very similar results and again a representative example is shown. Sequence reads were displayed on the UCSC genome browser. The presence of the S-DHS DNase-seq peak was verified in T47D cells (Figure S4). For DNase-chip the generation of libraries of DNase hypersensitive fragments has previously been described [22]. Libraries were hybridised to Agilent custom tiling arrays covering 2Mb at 8q24.

### Chromatin conformation capture (3C)

The 3C method [38] was applied to detect physical interactions between genomic regions (such as between promoters and distant enhancers [39]) using a sequence of interest (bait) from 8q24 region. The experimental procedure for this technique is outlined in Figure S12. The 3C experiments were carried out in the normal-like cell lines HB2 and RWPE-1 as many cancer cell lines such as PC3 and MCF-7 carry amplifications across the 8q24 region [9], [10]. Briefly, exponentially growing cells were cross-linked with 1% formaldehyde for 10 min at room temperature, washed 3x with cold PBS and scraped into microcentrifuge tubes. Cells were spun at 3000 rpm for 5 mins and resuspended in 1 ml lysis buffer (50 mM Tris-HCl pH 8.0, 1% SDS, 10 mM EDTA). After 10 min cells were centrifuged and resuspended in lysis buffer supplemented with 0.4% NP-40 and 1.8% Triton-X. After centrifugation for 20 seconds at full speed 1.5×10^6^ nuclei were resuspended in 300 µl *Eco*RI digestion buffer and 1.8% Triton-X. Nuclei were incubated for 45 minutes at 37°C in a cell shaker and subsequently digested with 1000 U *Eco*RI (NEB) overnight. The enzyme was heat-inactivated and the sample diluted in 1 ml. Ligation was carried out overnight using 4000 U T4 ligase at 16°C. A second digestion step was carried out using 1000 U *Bam*HI (NEB). Genomic DNA was purified after proteinase K treatment using standard protocols. PCRs were carried out using Power SYBR Green Mastermix (Applied Biosystems), 100 ng template DNA, 5 pmol of each primer in a volume of 20 µl (initial 95°C denaturation step, then 1 min at 60°C and 20 secs at 92°C for 40 cycles). Products were separated on a 3% NuSieve agarose gel. The identity of all PCR products was verified by direct sequencing of PCR products.

### Electrophoretic mobility shift assay (EMSA)

EMSAs were carried out as previously described [40]. Oligonucleotide sequences used in the assays are provided in Table S1. Antibodies for supershifts were obtained from Santa Cruz Biotech: αYY1 (sc1703x), αC/EBPα (sc9314x), αSP1 (sc14027x) and αOct-1 (sc232x) and 2 µl were used per reaction.

### Chromatin Immunoprecipitation (ChIPs)

ChIP reactions were carried out as described by Schmidt *et al*. [41] using 10 µg αYY1 (sc7341x, Santa Cruz Biotech). DNA was purified from the precipitates and used in RT-PCR reactions with primers listed in Table S1. Primer design was carried out using the UCSC genome browser mapability plot (Figure S3B) in conjunction with Primer 3 software. The primer pairs used gave rise to a single reproducible band whose identity was confirmed by sequencing. The RT-PCR protocol includes a dissociation step to confirm that the reaction gave rise to only a single product. Results were normalised against input and normal mouse IgG (sc2025, Santa Cruz Biotech). As positive control the promoter region of the glucocorticoid receptor [42] was chosen, as this contains three YY1 sites. Results are given relative to S-DHS-ve, a sequence 5 kb upstream of the S-DHS which we have shown to be in inaccessible chromatin (Figure 1). 1542-CP cells and MCF-7 cells are heterozygous for the risk SNP rs378854. Consistent results were obtained in three biological repeats. Error bars denote the technical error in a representative experiment.

### Cloning and transfection

S-DHS_cloning-forward and reverse primers (Table S1) were used to amplify a DNA fragment from genomic DNA of PMC42 breast cancer cell line (heterozygous for rs378854) and cloned using the TopoTA cloning system (Invitrogen). Plasmids were sequenced and clones carrying either the minor or the common allele of rs378854 were selected. The inserts were excised using *Sal*I and *Bam*HI (resulting fragment: chr8:128,392,958-128,393,353) and cloned into pGL3-basic or pGL3-enhancer vector (Promega), cut with *Xho*I and *Bgl*II. The same fragments were inserted into pGL3-promoter vector (Promega) cut with *Bam*HI and *Sal*I. Plasmids were prepared with an endotoxin-free kit (Qiagen) and transiently transfected into PC3 and MCF-7 cells grown in 24 well plates. 0.5 µg reporter plasmid and 0.1 µg β-galactoside transfection control plasmid in 30 µl OptiMEM (Gibco) were incubated for 20 min with 4.5 µl Fugene (Roche) before being added to 50% confluent wells. 10% FCS was added after 2 hours. Cells were harvested in 100 µl lysis buffer after 24 hours and luciferase and β-galatosidase activity was assayed using Promega kits. The data is averaged from two independent transfection experiments, each carried out in triplicate.

### Ethics statement and clinical data

Anonymised tumour and normal adjacent to tumour prostate tissue samples were either received from the National Cancer Institute (NCI) Cooperative Prostate Cancer Tissue Resource (CPCTR) or from the University of Maryland under protocols reviewed and approved by Institutional Review Boards. We also obtained clinical information including age at diagnosis, race, Gleason score and PSA level at diagnosis.

### Analysis of gene expression in prostate samples

The frozen samples were homogenized with Tissue Lyser (Qiagen) and divided into two fractions from which total RNA was prepared with MirVana kit (Applied Biosystems) and DNA was prepared with Gentra (Qiagen). The integrity of RNA was confirmed by Bioanalyzer (Agilent).

### mRNA expression analysis

cDNA was prepared from 800 ng of total RNA with Superscript III kit and random hexamers (Invitrogen). All expression assays were first evaluated in pooled prostate cDNA samples containing 25 ng, 5 ng, or 1 ng of total RNA per reaction. Genomic DNA and water were used as negative controls for each assay. Based on this test, endogenous controls Beta-2 microglobulin (*B2M*, assay HS_00187842_m1), Cyclophilin (*PPIA, assay* 4326316E) and *MYC* expression (assay Hs00905030) were tested using 5 ng of total RNA per reaction, while *PVT1* expression (assay Hs00413039) was evaluated with 20 ng of total RNA per reaction. Expression of alternative splicing forms of *PVT1* measured by assay Hs01069044 was beyond confident detection level even with 20 ng of total RNA and was not used on individual tissue samples.

The expression was tested in 4 technical replicates for each sample and assay, technical replicates were averaged and standard deviations were determined.

### miRNA expression

TaqMan miRNA expression assays for target miRNAs from *PVT1* gene (002872 for hsa-miR-1204, 002778 for hsa-miR-1205, 002878 for hsa-miR-1206, 002826 for hsa-miR-1207-3p, 241060-mat for hsa-miR-1207-5p, 002880 for hsa-mir-1208) and 3 endogenous controls (001973 for U6 snRNA, 001094 for RNU44 and 001006 for RNU48) as well as other reagents for miRNA expression were purchased from Applied Biosystems. cDNA was prepared from experimentally determined amount of total RNA with TaqMan MicroRNA reverse transcription kit. All assays were first tested on pooled cDNA samples using 10, 20 and 100 ng of total RNA per reaction as input. Based on this test, U6, RNU44 and RNU48 assays were run on all individual prostate samples starting from 20 ng of total RNA per reaction. For hsa-miR-1204, 1205, 1206 and 1207-3p reactions were run starting from 100 ng of total RNA per reaction. Expression of hsa-miR-1207-5p was below detection level and was not tested further. All expression assays were run in technical duplicates and target assays were normalized by a geometric mean of U6, RNU44 and RNU48.

### CNV analysis in *PVT1* region

A custom-designed assay was used to quantify copy number variation (CNV) within intron 1 of *PVT1* gene (PVT1-CNV-F and PVT1-CNV-R, 173 bp) (Table S1). The assay was run on DNA prepared from the prostate tissue samples with 5 ng of DNA, 2x Power SYBR Green master mix (Applied Biosystems) and primers. The results were normalized to copy number of control RNaseP assay quantified in the same DNA samples. All assays were run in four technical replicates on 7900 Sequence Detection System (Applied Biosystems).

### Statistical analysis

Statistical significance of differences in the reporter assays was determined using a Student's two-sided t-test with a Bonferroni correction for multiple comparisons. The expression values of mRNA and miRNA target assays were normalized by corresponding endogenous controls according to dCt method of relative quantification and tested for normality of distribution. Univariate linear regression was used to analyze expression values in relation to 0, 1 or 2 copies of risk alleles of rs378854. Age, race and *PVT1*-CNV values were tested as possible covariates but were found to have no significant effect. The analysis was performed with SPSS 16.0. Normalized expression values were centered to the mean of the samples heterozygous for rs378854 and plotted with GraphPad Prism5 software.

## Supporting Information

Figure S1 Map of the haplotype blocks (http://hapmap.ncbi.nlm.nih.gov) extending from the *FAM84B* to the *MYC* gene. Haplotype blocks that are associated with cancer are highlighted in colour. There are eight prostate cancer-associated loci (blue), one associated with prostate, colorectal and ovarian cancer (red) and one each for lymphoma (orange), breast cancer (green) and bladder cancer (purple). The position of two most strongly cancer-associated SNPs in the breast and prostate cancer risk locus examined in this study are shown by arrows.
(PPT)

Figure S2 Comparison of DNase I hypersensitivity of the 8q24 locus using Illumina sequencing (DNase-seq) for MCF-7 cells versus analysis by hybridisation to microarrays (DNase-chip). The “breast” lane gives probability plots for the average values obtained using independent hybridisation of duplicates of three different breast cancer cell lines: MCF-7, T47D and PMC42. Microarray data was normalised and analysed by the ACME algorithm and combined probability plots are shown. 95% cut-offs and a sliding window of 500 bp were used. A 100 kb window overlapping (A) the breast cancer susceptibility region and (B) the *MYC* and *PVT1* promoters is shown. Some peaks such as the S-DHS overlap repetitive elements and are not apparent in the microarray analysis since repetitive sequences are not tiled (indicated by red arrows). Peaks that are apparent in microarray but not sequencing experiments are most likely due to cross-hybridisation with non-specific sequences on the microarray (exemplified by a blue arrow).
(PPT)

Figure S3 Examination of DNase-seq data for the region surrounding the S-DHS. (A) The DNase-seq tracks for chr8: 128,380,00–128,411,500 are shown for two independent experiments for each cell line examined, denoted a and b in each case. For each experiment the number of reads was adjusted to the total number of reads obtained in the experiment. The regions shown includes all SNPs with an r^2^>0.8 for rs620861 (see Figure S6). (B) DNase-seq tracks derived with two different stringency settings within the BWA software (map01 and map15) for MCF-7 and RWPE-1 for the LINE element that contains the S-DHS are shown. As the S-DHS maps to a LINE element, correct alignment of sequence reads to the reference genome is of critical importance. We therefore tested different stringency values in the alignment algorithms, but found that even when alignments of sequence reads are called with a confidence greater than 97% (map15), MCF-7 displays a strong signal. The LINE element overlapping the S-DHS contains sufficient information to allow unique assignment of sequence reads as indicated by the “mapability track” on the UCSC genome browser. This track (Duke uniqueness for 35 bp) depicts those regions in the genome where unique alignments of sequence reads to the reference genome are possible, allowing for 2 mismatches. The alignment peaks in the sequencing track are wider than those in the mapability track since 44mers were determined in the Illumina sequencing. When shorter oligomers are examined (Umass Uniq15) fewer of the peaks can be uniquely assigned to the reference genome. The LINE element is not marked by the ENCODE artefacts track, suggesting that standard sequence alignment algorithms can be used for these regions. The “Human Chained Self Alignments”-track indicates that the LINE element under investigation self chains only to a single LINE element on chromosome 3.
(PPT)

Figure S4 Analysis of properties of the two highly homologous LINE elements on chromosome 8 (overlapping the S-DHS) and chromosome 3. DHS-seq tracks for MCF-7 and RWPE-1, mapped with an alignment stringency of 15, are shown for (A) chr8: 128,392,900–128,396,800 and (B) chr3: 11,505,153–11,509,023. The sequences on chromosome 3 are shown in the opposite direction with respect to the reference genome. The highly similar LINE element on chromosome 3 also shows some DNase I hypersensitivity in MCF-7 cells, but the signal is 3-fold lower, making it highly unlikely that the S-DHS observed on chromosome 8 is due to a “spill-over” of sequence read alignments from chromosome 3.
(PPT)

Figure S5 Chromatin immunoprecipitation (ChIP) assay for the breast and prostate cancer susceptibility region in MCF-7 cells. All experiments were carried out by ChIP-seq [24] and results are shown for the two cohesin subunits Rad21 and SA1, CTCF and FoxA1. The peaks obtained overlap with the S-DHS identified here. (A) depicts the breast cancer susceptibility region (chr8:128,380,000–128,460,000), while (B) shows an enlarged view of the S-DHS. Each panel also shows UCSC mapability plots (Duke Unique 35) and repeat elements across the region. As for the DNase-seq, alignment peaks occur only at those sequences that are sufficiently unique to allow mapping to the genome. Unmappable regions between peaks may be occupied, but short read sequencing is not informative for these regions.
(PPT)

Figure S6 The haplotype block encompassing both the prostate and breast cancer susceptibility hits is shown. Blue arrows correspond to the 14 variants that are correlated with rs620861, the top hit identified by Al Olama et al. [3] at r^2^>0.8. Green arrows highlight the variants strongly correlated with rs13281615, the top hit for breast cancer susceptibility [2], again at r^2^>0.8. The two sets of SNPs cluster to different region of the haplotype block.
(PPT)

Figure S7 (A) DNase-seq trace for MCF-7 and T47D of a 1.2 kb fragment overlapping the S-DHS. The position of rs378854 and the relative position of the fragment used in functional assays are shown. (B) SNPs identified within this 1.2 kb fragment. Chromosome position, minor allele frequency (MAF), alleles and correlation to the top hit prostate cancer SNP rs620861 are given.
(PPT)

Figure S8 Analysis of copy number variation (CNV) within the *PVT1* region. (A) The mean values and 95% confidence intervals for *PVT1*_*CNV* normalized by *RNAseP* values are given for groups of normal and tumour prostate tissue samples. CNV_*PVT1* values for each sample were calculated as CNV  =  Ct (*RNAseP*) –Ct (*PVT1*); each Ct value was measured in 4 technical replicates and plotted on a Log2 scale. Ct - is a PCR cycle of detection of the signal by qPCR. First, we tested if *PVT1_CNV* was different in normal and tumor samples, but detected no significant difference (p = 0.849). (B) *PVT1* mRNA expression after adjustment by *PVT1_CNV* and in relation to the number of risk alleles of rs378854 in 53 normal prostate tissue samples for which both mRNA expression and *PVT1_CNV* information was available. The *PVT1*_CNV did not significantly affect the results for *PVT1* mRNA expression: effect of *PVT1_CNV*, p = 0.708; effect of rs378854, p = 0.047 (adjusted for *PVT1_CNV*); effect of rs378854, p = 0.025 (not adjusted for *PVT1_CNV*, in 59 normal prostate samples). Thus, the effect of rs378854 remains significant (p = 0.047) even after adjustment by *PVT1_CNV* values.
(PPT)

Figure S9 Quantitative expression for hsa-mir-1208 in normal and tumour prostate tissue samples. The expression has been normalized by a geometric mean of U6, RNU44 and RNU48 endogenous controls and is presented in log2 scale. The expression in carriers of 0 or 1 risk alleles of rs378854 is comparable in normal and tumour tissue samples, while in samples homozygous for risk allele, the expression is increased in normal samples while it's decreased in tumours, providing a significant interaction effect (p = 0.020).
(PPT)

Figure S10 Chromatin accessibility of the 1 kb region (chr8: 128,482,000–128,483,000) containing the colon and prostate-specific enhancer marked by rs6983267 (chr8: 128,482,487). DNase-chip data are shown for three breast cancer cell lines (MCF-7, T47D and PMC42 =  breast), two prostate cell lines (LnCap and RWPE-1 =  prostate) and the HCT116 ( = colon) cell line. Microarray data was normalised and analysed by the ACME algorithm and combined probability plots are shown. 95% cut-offs and a sliding window of 500 bp were used in the ACME algorithm.
(PPT)

Figure S11 Cartoon showing the cancer predisposition loci in a 3.2 Mb genomic interval on 8q24, spanning the nearest expressed neighbours of the *MYC* and *PVT1* genes. Grey boxes depict RefSeq genes, with light grey indicating no expression in prostate cells. Ovals depict cancer predisposition hits with tissue subtypes as indicated by the colours (UCSC genome browser and [36]).
(PPT)

Figure S12 Carto on of Chromatin Conformation Assay.
(PPT)

Table S1 Oligonucleotides used in this study.
(DOC)
