# Supplementary figures and images for: A Functional Variant at a Prostate Cancer Predisposition Locus at 8q24 Is Associated with PVT1 Expression
Source: PLoS Genet. 2011 Jul 21;7(7):e1002165. doi: 10.1371/journal.pgen.1002165 (PMC3140991; doi:10.1371/journal.pgen.1002165)

## Slide 1
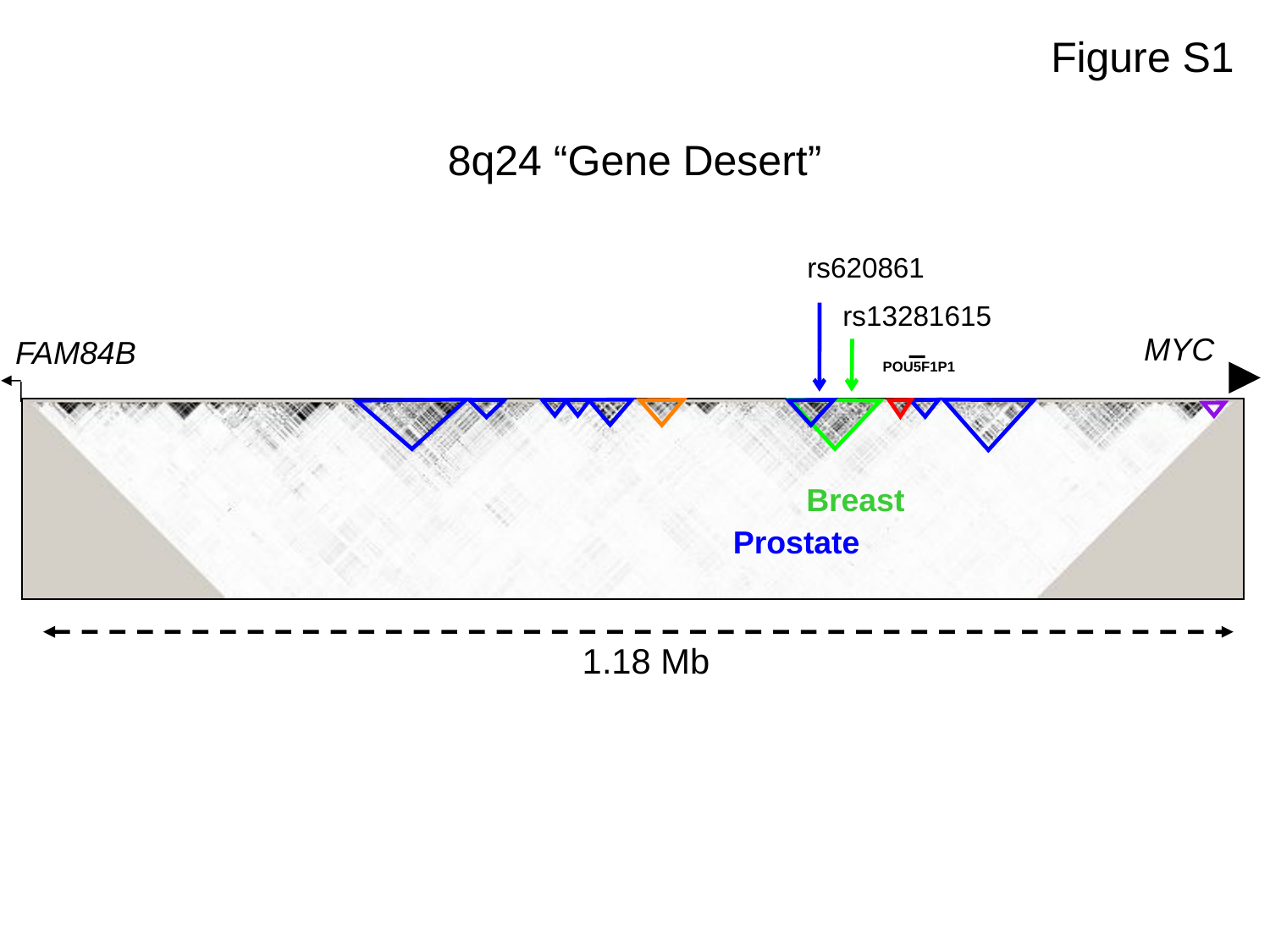

Figure S1
8q24 “Gene Desert”
rs620861
rs13281615
MYC
FAM84B
POU5F1P1
Breast
Prostate
1.18 Mb

Supplement: Figure S1 — Map of the haplotype blocks (http://hapmap.ncbi.nlm.nih.gov) extending from the FAM84B to the MYC gene. Haplotype blocks that are associated with cancer are highlighted in colour. There are eight prostate cancer-associated loci (blue), one associated with prostate, colorectal and ovarian cancer (red) and one each for lymphoma (orange), breast cancer (green) and bladder cancer (purple). The position of two most strongly cancer-associated SNPs in the breast and prostate cancer risk locus examined in this study are shown by arrows. (PPT) [file pgen.1002165.s001.ppt]

## Slide 1
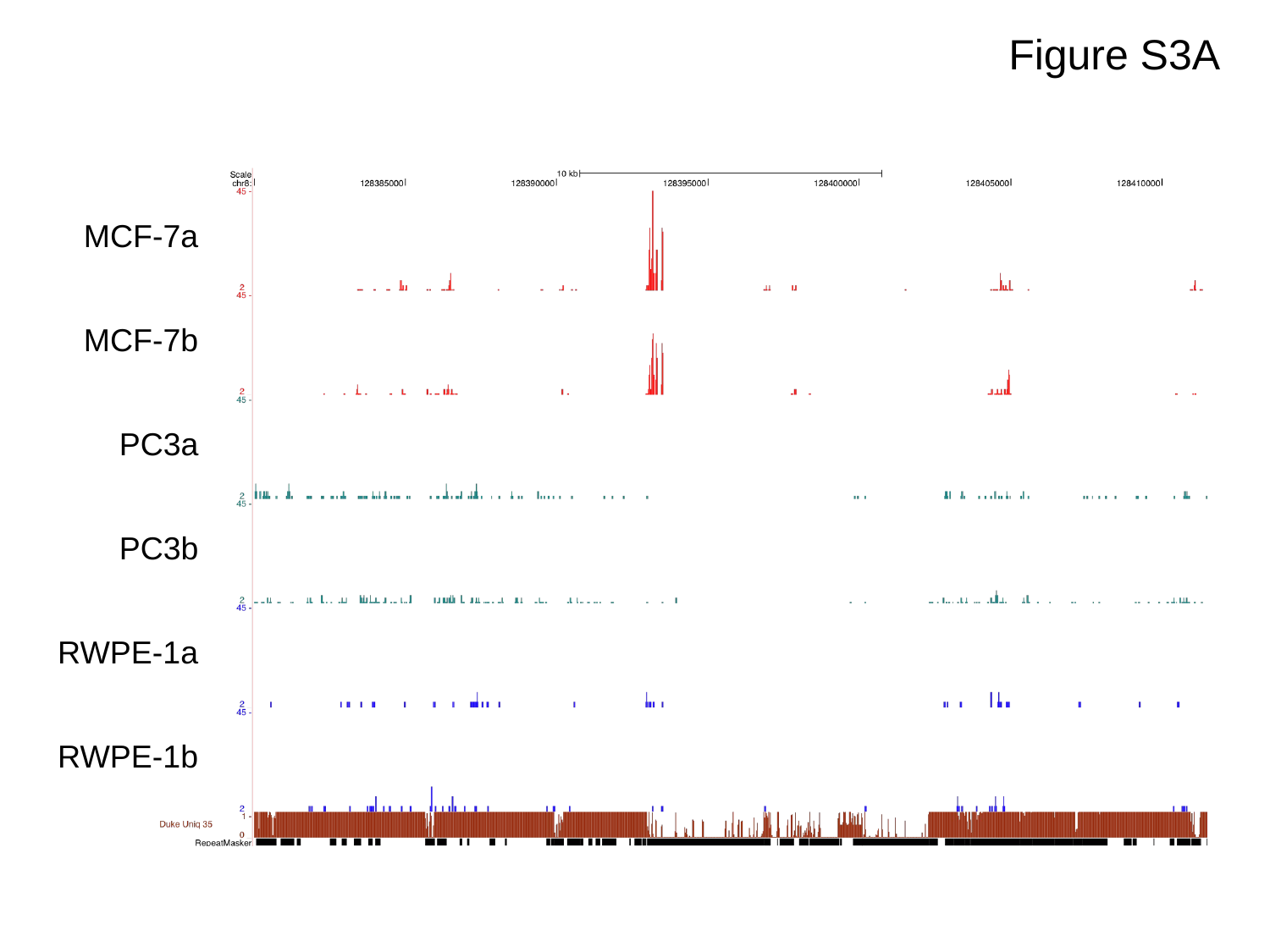

Figure S3A
MCF-7a
MCF-7b
PC3a
PC3b
RWPE-1a
RWPE-1b

## Slide 2
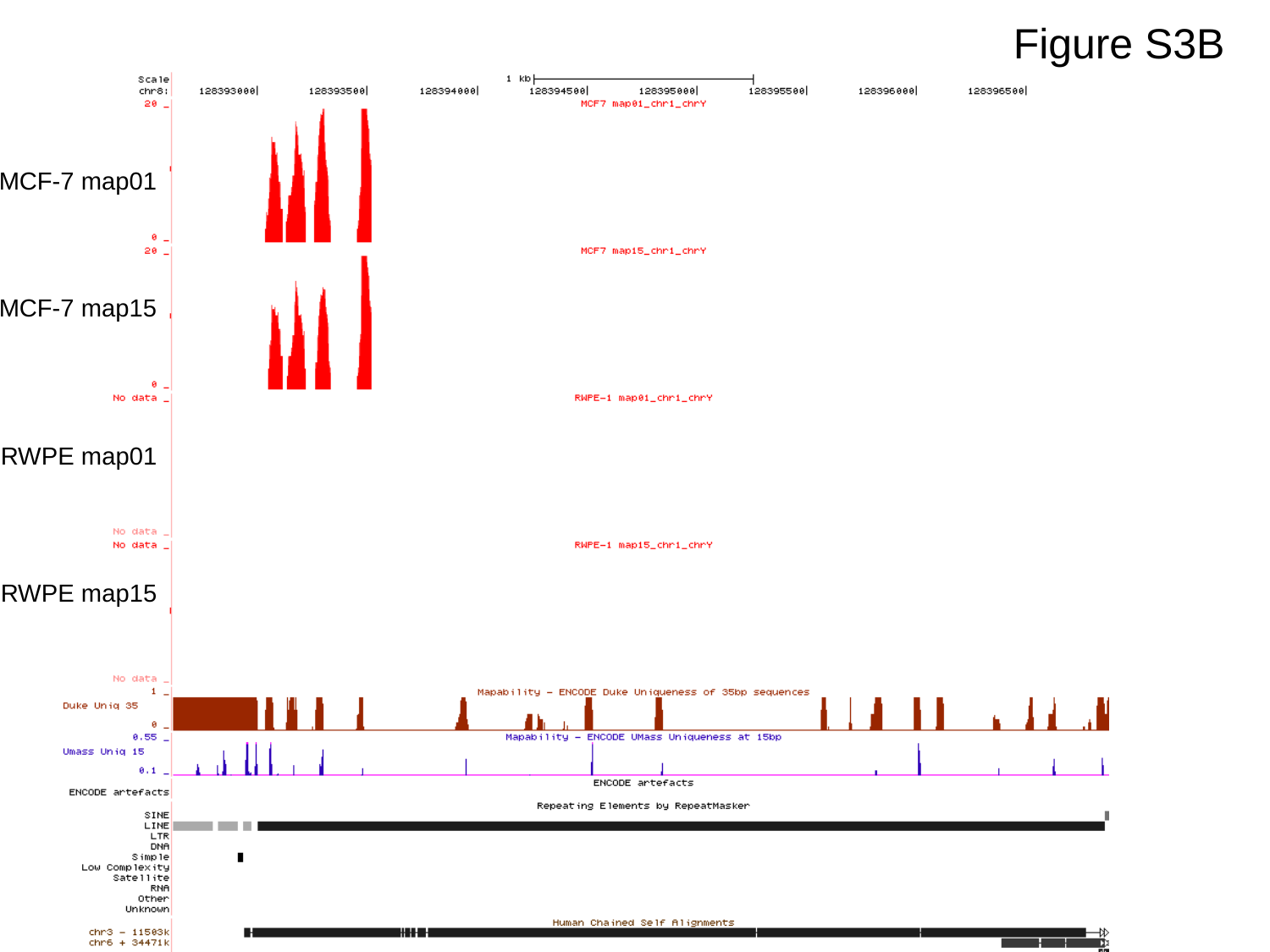

Figure S3B
MCF-7 map01
MCF-7 map15
RWPE map01
RWPE map15

Supplement: Figure S3 — Examination of DNase-seq data for the region surrounding the S-DHS. (A) The DNase-seq tracks for chr8: 128,380,00–128,411,500 are shown for two independent experiments for each cell line examined, denoted a and b in each case. For each experiment the number of reads was adjusted to the total number of reads obtained in the experiment. The regions shown includes all SNPs with an r2>0.8 for rs620861 (see Figure S6). (B) DNase-seq tracks derived with two different stringency settings within the BWA software (map01 and map15) for MCF-7 and RWPE-1 for the LINE element that contains the S-DHS are shown. As the S-DHS maps to a LINE element, correct alignment of sequence reads to the reference genome is of critical importance. We therefore tested different stringency values in the alignment algorithms, but found that even when alignments of sequence reads are called with a confidence greater than 97% (map15), MCF-7 displays a strong signal. The LINE element overlapping the S-DHS contains sufficient information to allow unique assignment of sequence reads as indicated by the “mapability track” on the UCSC genome browser. This track (Duke uniqueness for 35 bp) depicts those regions in the genome where unique alignments of sequence reads to the reference genome are possible, allowing for 2 mismatches. The alignment peaks in the sequencing track are wider than those in the mapability track since 44mers were determined in the Illumina sequencing. When shorter oligomers are examined (Umass Uniq15) fewer of the peaks can be uniquely assigned to the reference genome. The LINE element is not marked by the ENCODE artefacts track, suggesting that standard sequence alignment algorithms can be used for these regions. The “Human Chained Self Alignments”-track indicates that the LINE element under investigation self chains only to a single LINE element on chromosome 3. (PPT) [file pgen.1002165.s003.ppt]

## Slide 1
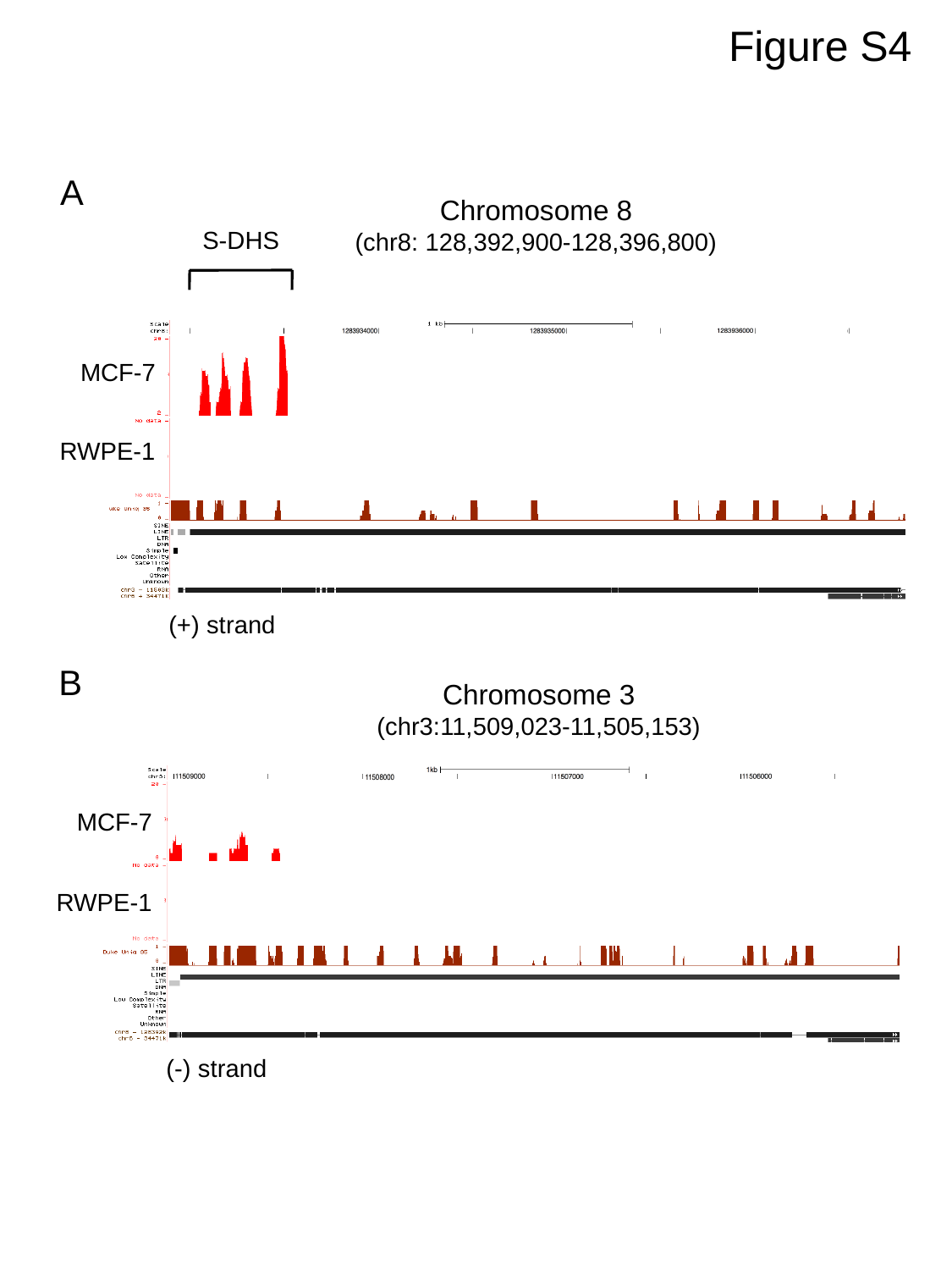

Figure S4
A
Chromosome 8
(chr8: 128,392,900-128,396,800)
S-DHS
MCF-7
RWPE-1
(+) strand
B
Chromosome 3
(chr3:11,509,023-11,505,153)
MCF-7
RWPE-1
(-) strand

Supplement: Figure S4 — Analysis of properties of the two highly homologous LINE elements on chromosome 8 (overlapping the S-DHS) and chromosome 3. DHS-seq tracks for MCF-7 and RWPE-1, mapped with an alignment stringency of 15, are shown for (A) chr8: 128,392,900–128,396,800 and (B) chr3: 11,505,153–11,509,023. The sequences on chromosome 3 are shown in the opposite direction with respect to the reference genome. The highly similar LINE element on chromosome 3 also shows some DNase I hypersensitivity in MCF-7 cells, but the signal is 3-fold lower, making it highly unlikely that the S-DHS observed on chromosome 8 is due to a “spill-over” of sequence read alignments from chromosome 3. (PPT) [file pgen.1002165.s004.ppt]

## Slide 1
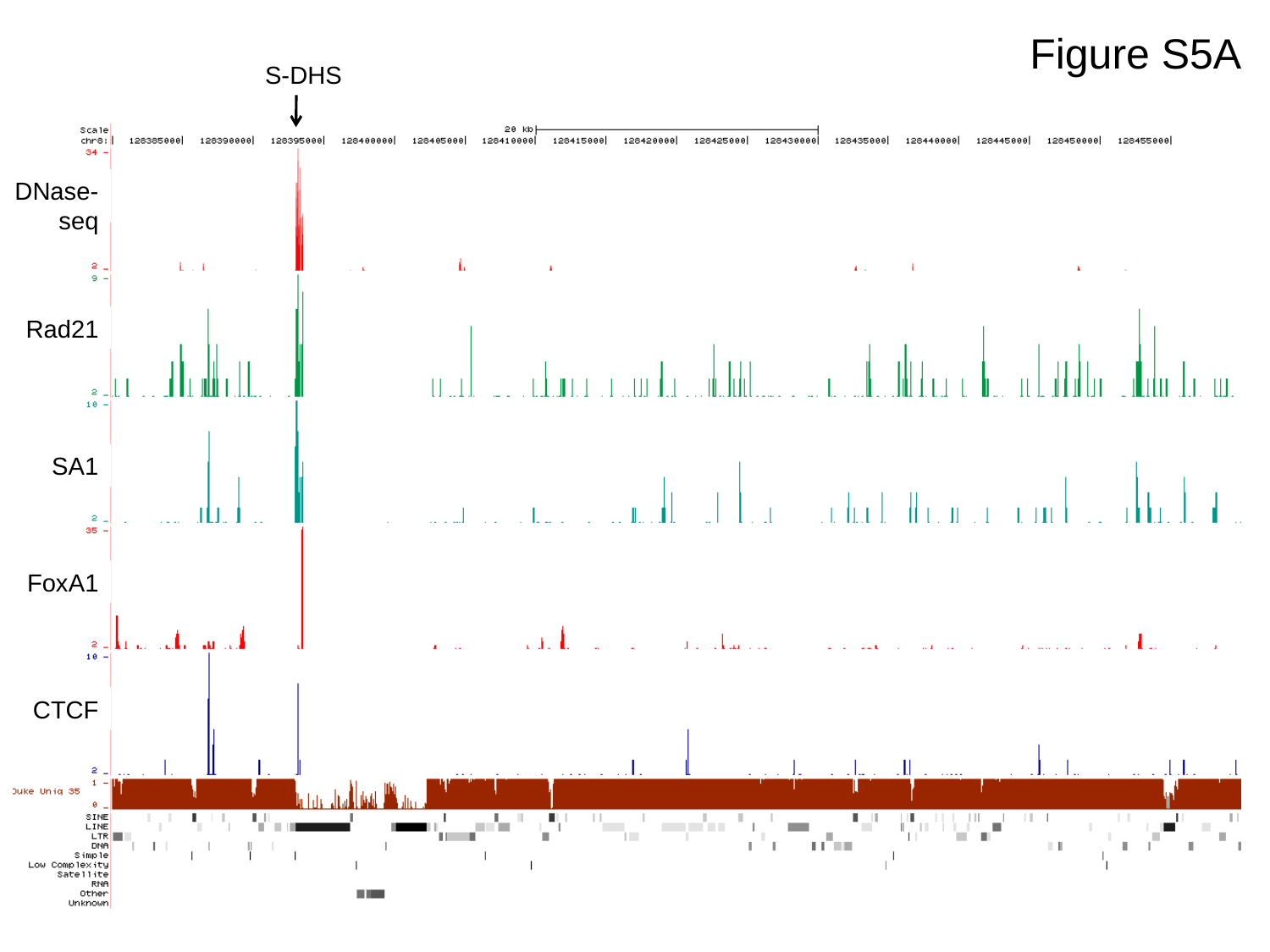

Figure S5A
S-DHS
DNase-seq
Rad21
SA1
FoxA1
CTCF

## Slide 2
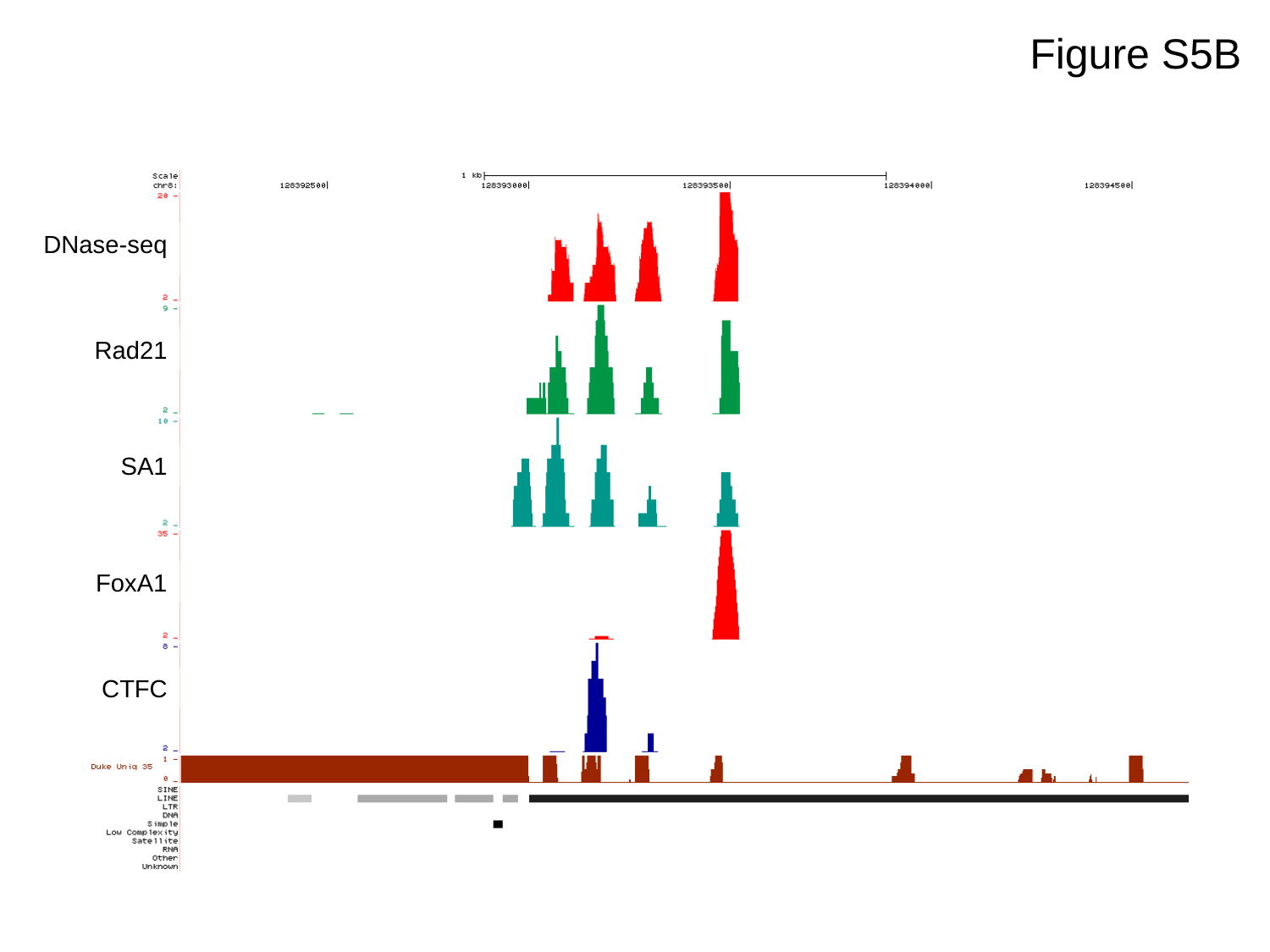

Figure S5B
DNase-seq
Rad21
SA1
FoxA1
CTFC

Supplement: Figure S5 — Chromatin immunoprecipitation (ChIP) assay for the breast and prostate cancer susceptibility region in MCF-7 cells. All experiments were carried out by ChIP-seq [24] and results are shown for the two cohesin subunits Rad21 and SA1, CTCF and FoxA1. The peaks obtained overlap with the S-DHS identified here. (A) depicts the breast cancer susceptibility region (chr8:128,380,000–128,460,000), while (B) shows an enlarged view of the S-DHS. Each panel also shows UCSC mapability plots (Duke Unique 35) and repeat elements across the region. As for the DNase-seq, alignment peaks occur only at those sequences that are sufficiently unique to allow mapping to the genome. Unmappable regions between peaks may be occupied, but short read sequencing is not informative for these regions. (PPT) [file pgen.1002165.s005.ppt]

## Slide 1
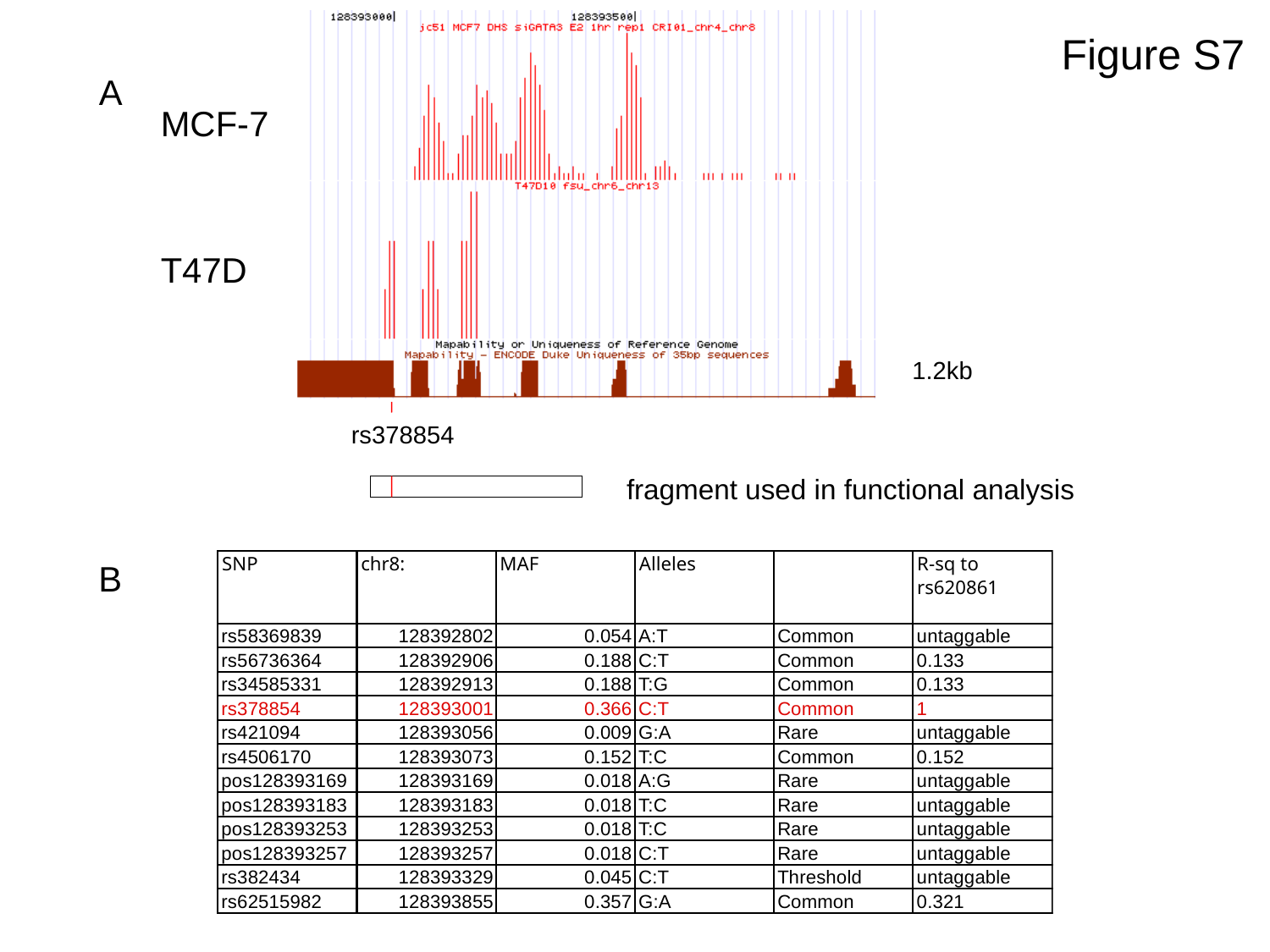

Figure S7
A
MCF-7
T47D
1.2kb
rs378854
fragment used in functional analysis
B

Supplement: Figure S7 — (A) DNase-seq trace for MCF-7 and T47D of a 1.2 kb fragment overlapping the S-DHS. The position of rs378854 and the relative position of the fragment used in functional assays are shown. (B) SNPs identified within this 1.2 kb fragment. Chromosome position, minor allele frequency (MAF), alleles and correlation to the top hit prostate cancer SNP rs620861 are given. (PPT) [file pgen.1002165.s007.ppt]

## Slide 1
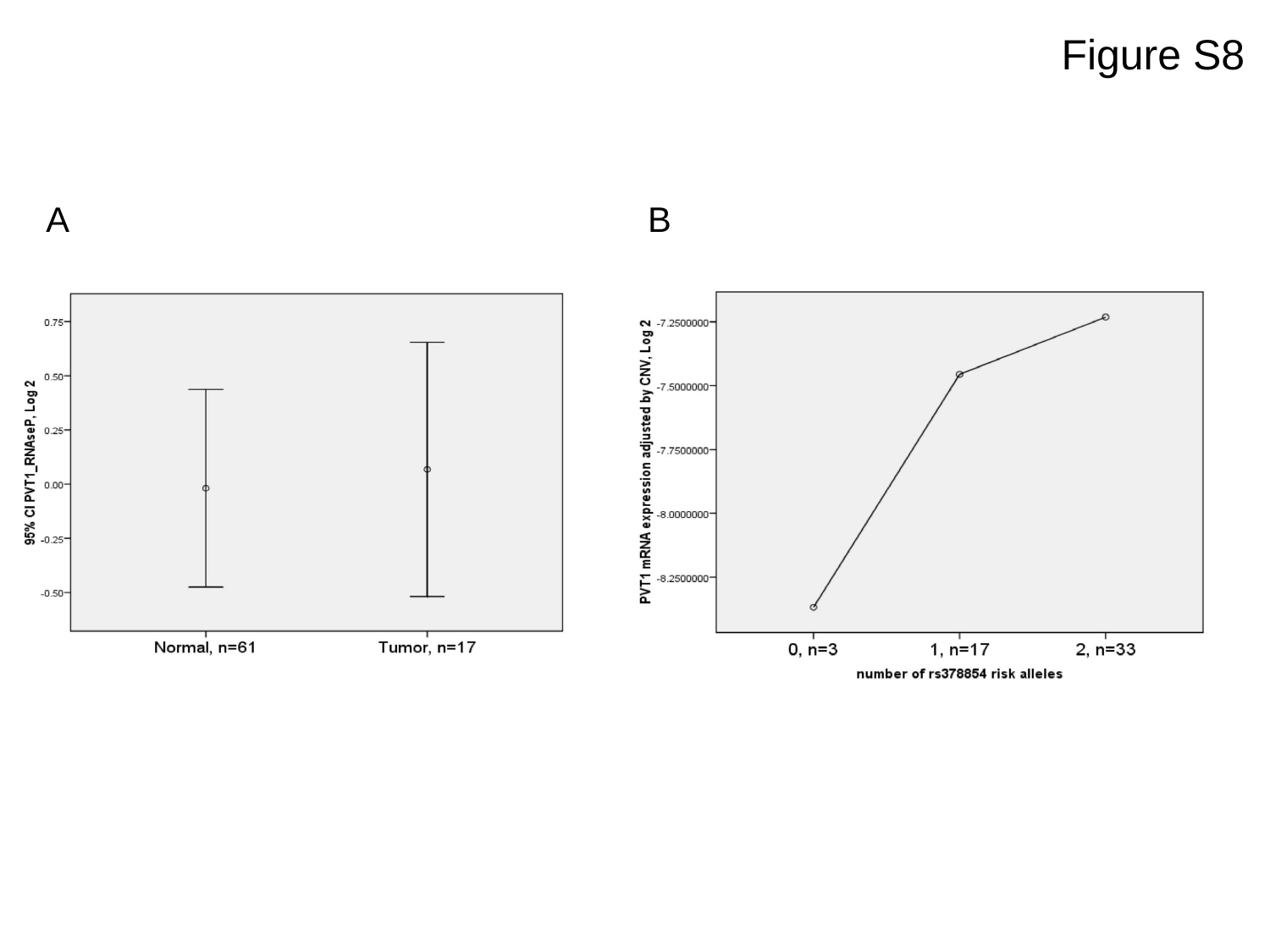

Figure S8
A
B

Supplement: Figure S8 — Analysis of copy number variation (CNV) within the PVT1 region. (A) The mean values and 95% confidence intervals for PVT1_CNV normalized by RNAseP values are given for groups of normal and tumour prostate tissue samples. CNV_PVT1 values for each sample were calculated as CNV = Ct (RNAseP) –Ct (PVT1); each Ct value was measured in 4 technical replicates and plotted on a Log2 scale. Ct - is a PCR cycle of detection of the signal by qPCR. First, we tested if PVT1_CNV was different in normal and tumor samples, but detected no significant difference (p = 0.849). (B) PVT1 mRNA expression after adjustment by PVT1_CNV and in relation to the number of risk alleles of rs378854 in 53 normal prostate tissue samples for which both mRNA expression and PVT1_CNV information was available. The PVT1_CNV did not significantly affect the results for PVT1 mRNA expression: effect of PVT1_CNV, p = 0.708; effect of rs378854, p = 0.047 (adjusted for PVT1_CNV); effect of rs378854, p = 0.025 (not adjusted for PVT1_CNV, in 59 normal prostate samples). Thus, the effect of rs378854 remains significant (p = 0.047) even after adjustment by PVT1_CNV values. (PPT) [file pgen.1002165.s008.ppt]

## Slide 1
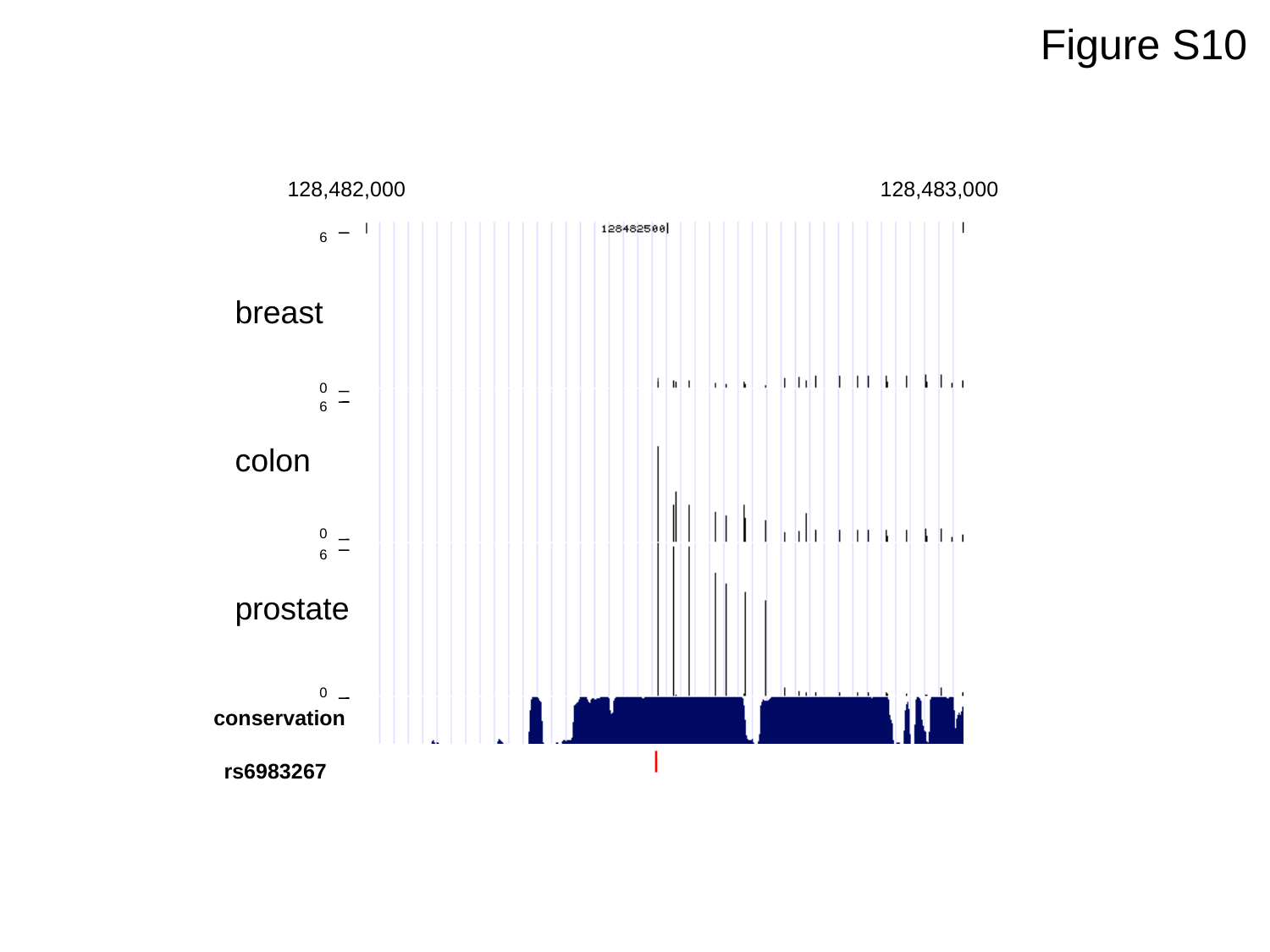

Figure S10
128,482,000
128,483,000
6
breast
0
6
colon
0
6
prostate
0
conservation
rs6983267

Supplement: Figure S10 — Chromatin accessibility of the 1 kb region (chr8: 128,482,000–128,483,000) containing the colon and prostate-specific enhancer marked by rs6983267 (chr8: 128,482,487). DNase-chip data are shown for three breast cancer cell lines (MCF-7, T47D and PMC42 = breast), two prostate cell lines (LnCap and RWPE-1 = prostate) and the HCT116 ( = colon) cell line. Microarray data was normalised and analysed by the ACME algorithm and combined probability plots are shown. 95% cut-offs and a sliding window of 500 bp were used in the ACME algorithm. (PPT) [file pgen.1002165.s010.ppt]
