# Supplementary material for: A Functional Variant at a Prostate Cancer Predisposition Locus at 8q24 Is Associated with PVT1 Expression
Source: PLoS Genet. 2011 Jul 21;7(7):e1002165. doi: 10.1371/journal.pgen.1002165 (PMC3140991; doi:10.1371/journal.pgen.1002165)
Supplement: Figure S2 — Comparison of DNase I hypersensitivity of the 8q24 locus using Illumina sequencing (DNase-seq) for MCF-7 cells versus analysis by hybridisation to microarrays (DNase-chip). The “breast” lane gives probability plots for the average values obtained using independent hybridisation of duplicates of three different breast cancer cell lines: MCF-7, T47D and PMC42. Microarray data was normalised and analysed by the ACME algorithm and combined probability plots are shown. 95% cut-offs and a sliding window of 500 bp were used. A 100 kb window overlapping (A) the breast cancer susceptibility region and (B) the MYC and PVT1 promoters is shown. Some peaks such as the S-DHS overlap repetitive elements and are not apparent in the microarray analysis since repetitive sequences are not tiled (indicated by red arrows). Peaks that are apparent in microarray but not sequencing experiments are most likely due to cross-hybridisation with non-specific sequences on the microarray (exemplified by a blue arrow). (PPT) [file pgen.1002165.s002.ppt]

## Slide 1
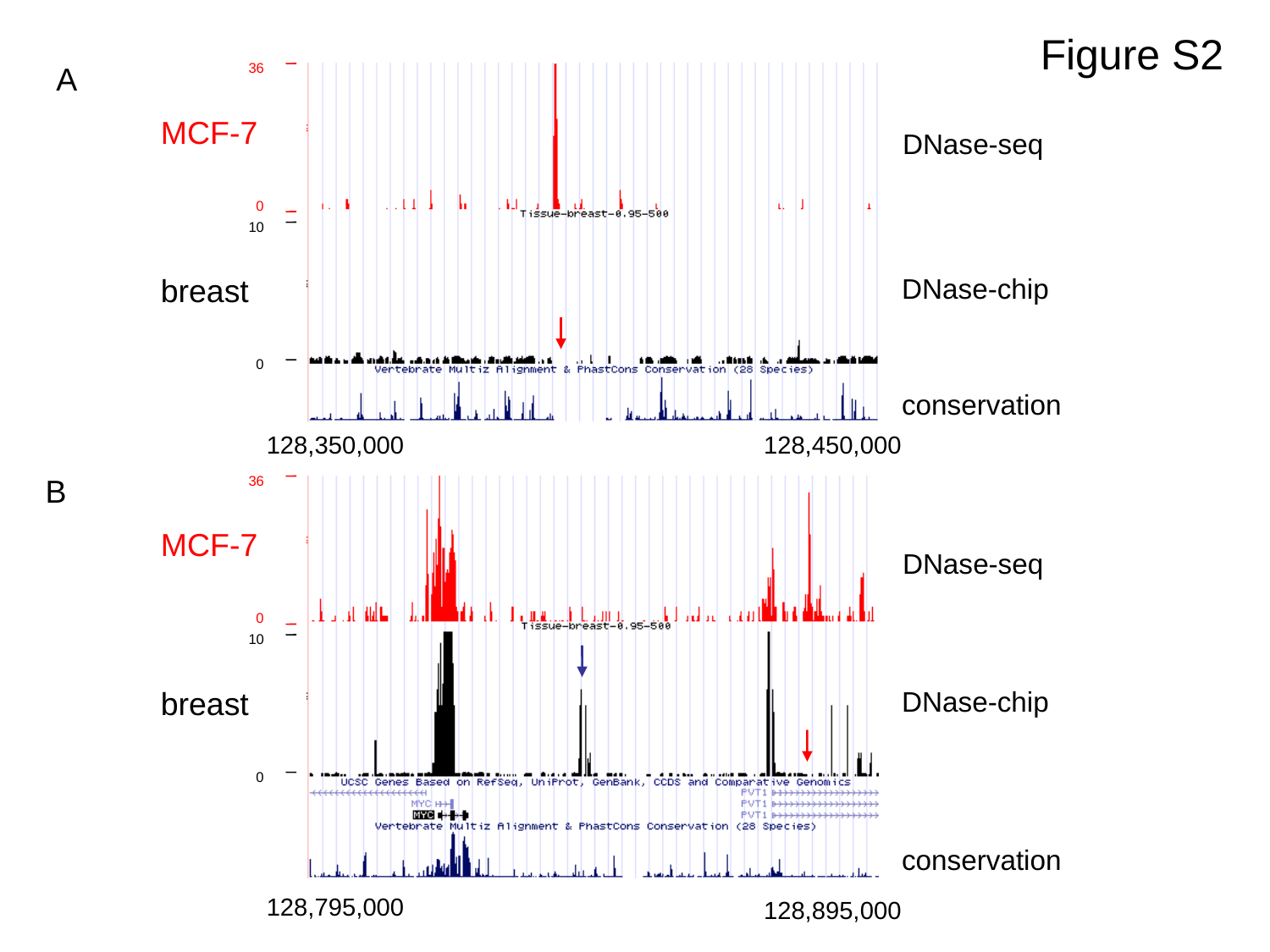

Figure S2
A
36
MCF-7
DNase-seq
0
10
breast
DNase-chip
0
conservation
128,350,000
128,450,000
B
36
MCF-7
DNase-seq
0
10
breast
DNase-chip
0
conservation
128,795,000
128,895,000
