# Supplementary material for: A Functional Variant at a Prostate Cancer Predisposition Locus at 8q24 Is Associated with PVT1 Expression
Source: PLoS Genet. 2011 Jul 21;7(7):e1002165. doi: 10.1371/journal.pgen.1002165 (PMC3140991; doi:10.1371/journal.pgen.1002165)
Supplement: Figure S6 — The haplotype block encompassing both the prostate and breast cancer susceptibility hits is shown. Blue arrows correspond to the 14 variants that are correlated with rs620861, the top hit identified by Al Olama et al. [3] at r2>0.8. Green arrows highlight the variants strongly correlated with rs13281615, the top hit for breast cancer susceptibility [2], again at r2>0.8. The two sets of SNPs cluster to different region of the haplotype block. (PPT) [file pgen.1002165.s006.ppt]

## Slide 1
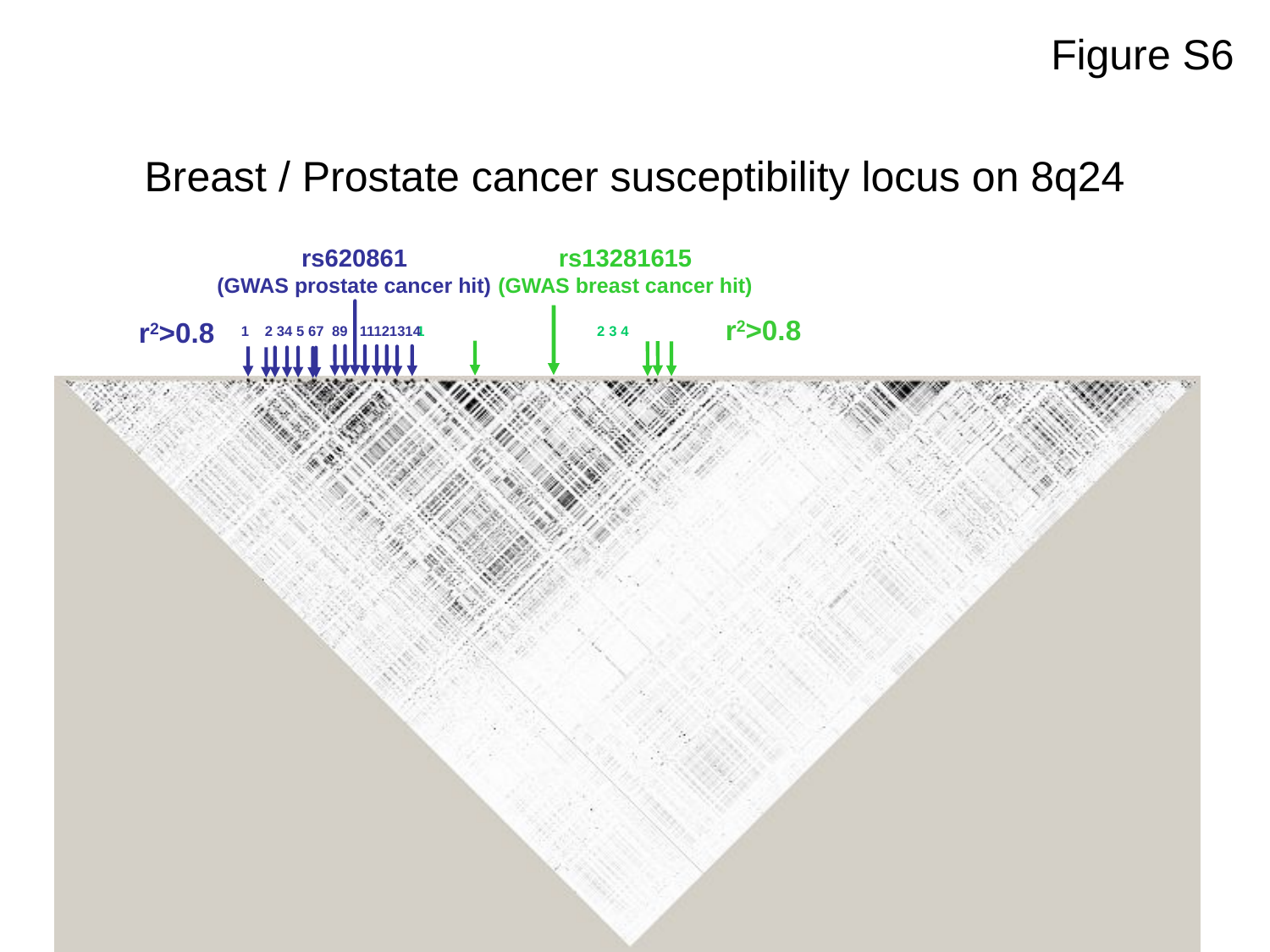

Figure S6
Breast / Prostate cancer susceptibility locus on 8q24
rs620861
(GWAS prostate cancer hit)
rs13281615
(GWAS breast cancer hit)
r2>0.8
r2>0.8
1 2 34 5 67 89 11121314
	1 2 3 4
