# Supplementary material for: A Functional Variant at a Prostate Cancer Predisposition Locus at 8q24 Is Associated with PVT1 Expression
Source: PLoS Genet. 2011 Jul 21;7(7):e1002165. doi: 10.1371/journal.pgen.1002165 (PMC3140991; doi:10.1371/journal.pgen.1002165)
Supplement: Figure S9 — Quantitative expression for hsa-mir-1208 in normal and tumour prostate tissue samples. The expression has been normalized by a geometric mean of U6, RNU44 and RNU48 endogenous controls and is presented in log2 scale. The expression in carriers of 0 or 1 risk alleles of rs378854 is comparable in normal and tumour tissue samples, while in samples homozygous for risk allele, the expression is increased in normal samples while it's decreased in tumours, providing a significant interaction effect (p = 0.020). (PPT) [file pgen.1002165.s009.ppt]

## Slide 1
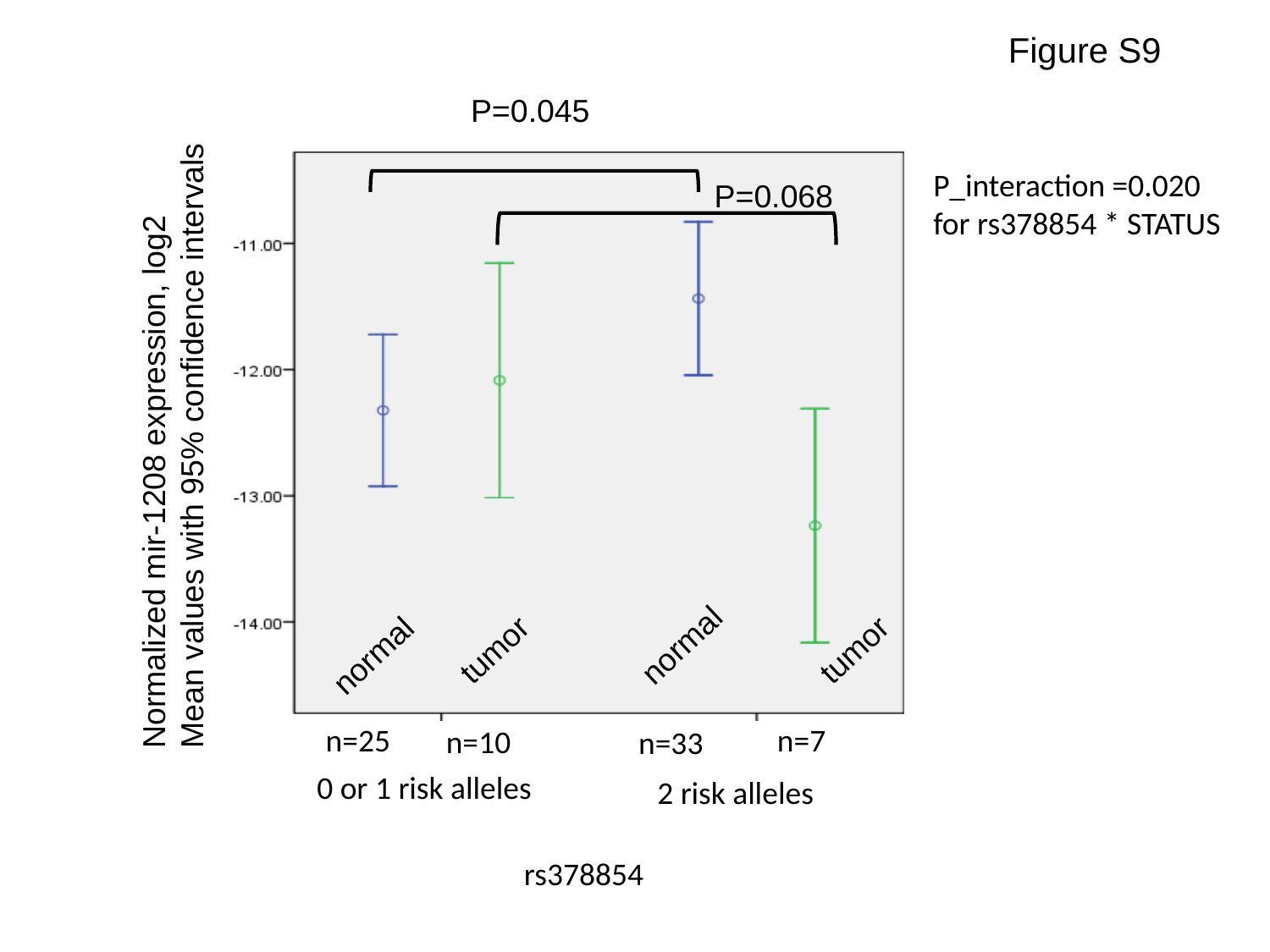

Figure S9
P=0.045
P_interaction =0.020
for rs378854 * STATUS
P=0.068
Normalized mir-1208 expression, log2
Mean values with 95% confidence intervals
normal
tumor
tumor
normal
n=25
n=7
n=10
n=33
0 or 1 risk alleles
2 risk alleles
rs378854
