# Supplementary material for: A Functional Variant at a Prostate Cancer Predisposition Locus at 8q24 Is Associated with PVT1 Expression
Source: PLoS Genet. 2011 Jul 21;7(7):e1002165. doi: 10.1371/journal.pgen.1002165 (PMC3140991; doi:10.1371/journal.pgen.1002165)
Supplement: Figure S11 — Cartoon showing the cancer predisposition loci in a 3.2 Mb genomic interval on 8q24, spanning the nearest expressed neighbours of the MYC and PVT1 genes. Grey boxes depict RefSeq genes, with light grey indicating no expression in prostate cells. Ovals depict cancer predisposition hits with tissue subtypes as indicated by the colours (UCSC genome browser and [36]). (PPT) [file pgen.1002165.s011.ppt]

## Slide 1
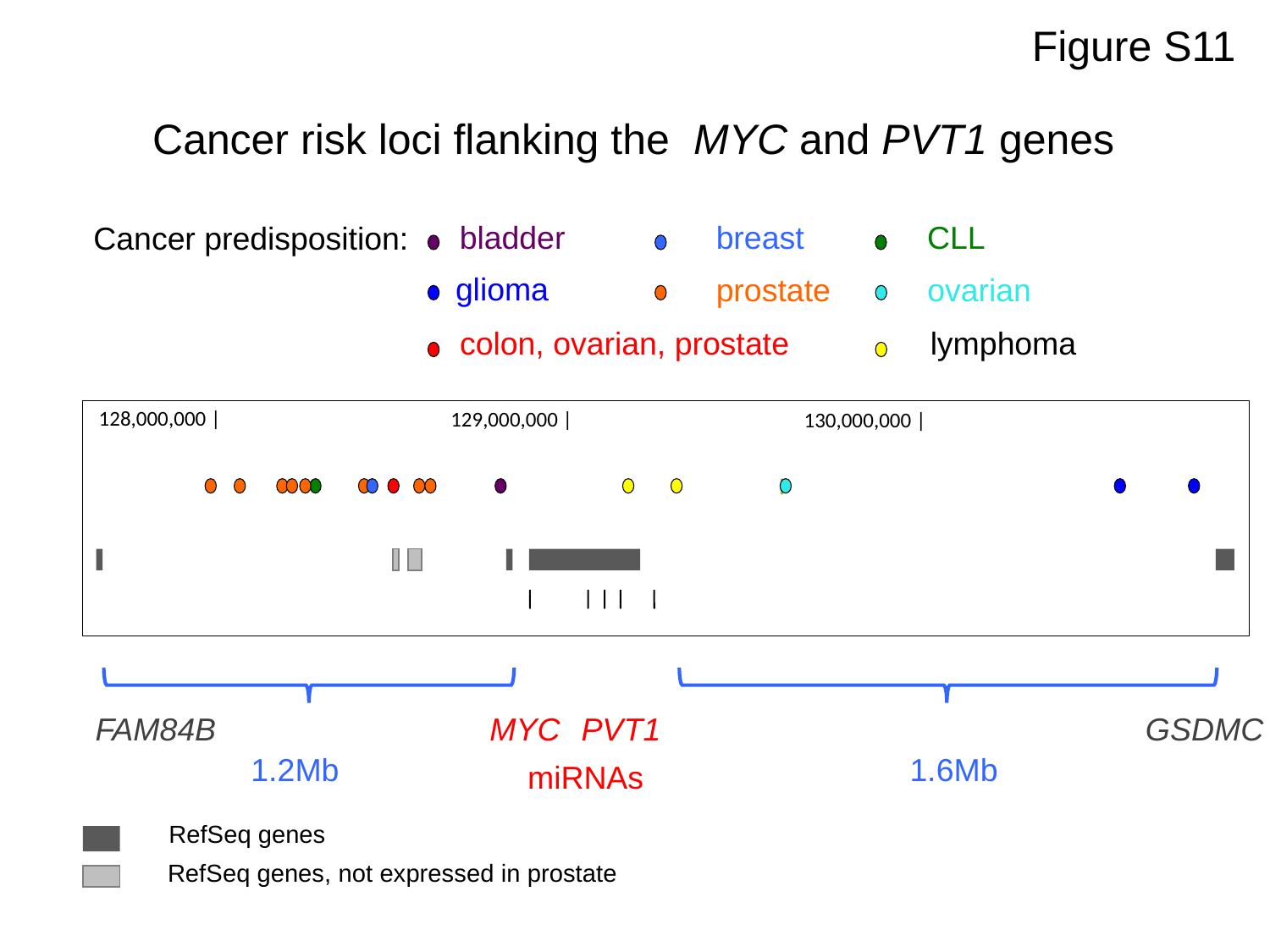

Figure S11
Cancer risk loci flanking the MYC and PVT1 genes
bladder
breast
CLL
Cancer predisposition:
glioma
prostate
ovarian
lymphoma
colon, ovarian, prostate
128,000,000 |
129,000,000 |
130,000,000 |
FAM84B
MYC
PVT1
GSDMC
1.2Mb
1.6Mb
miRNAs
RefSeq genes
RefSeq genes, not expressed in prostate
