# Supplementary material for: A Functional Variant at a Prostate Cancer Predisposition Locus at 8q24 Is Associated with PVT1 Expression
Source: PLoS Genet. 2011 Jul 21;7(7):e1002165. doi: 10.1371/journal.pgen.1002165 (PMC3140991; doi:10.1371/journal.pgen.1002165)
Supplement: Figure S12 — Carto on of Chromatin Conformation Assay. (PPT) [file pgen.1002165.s012.ppt]

## Slide 1
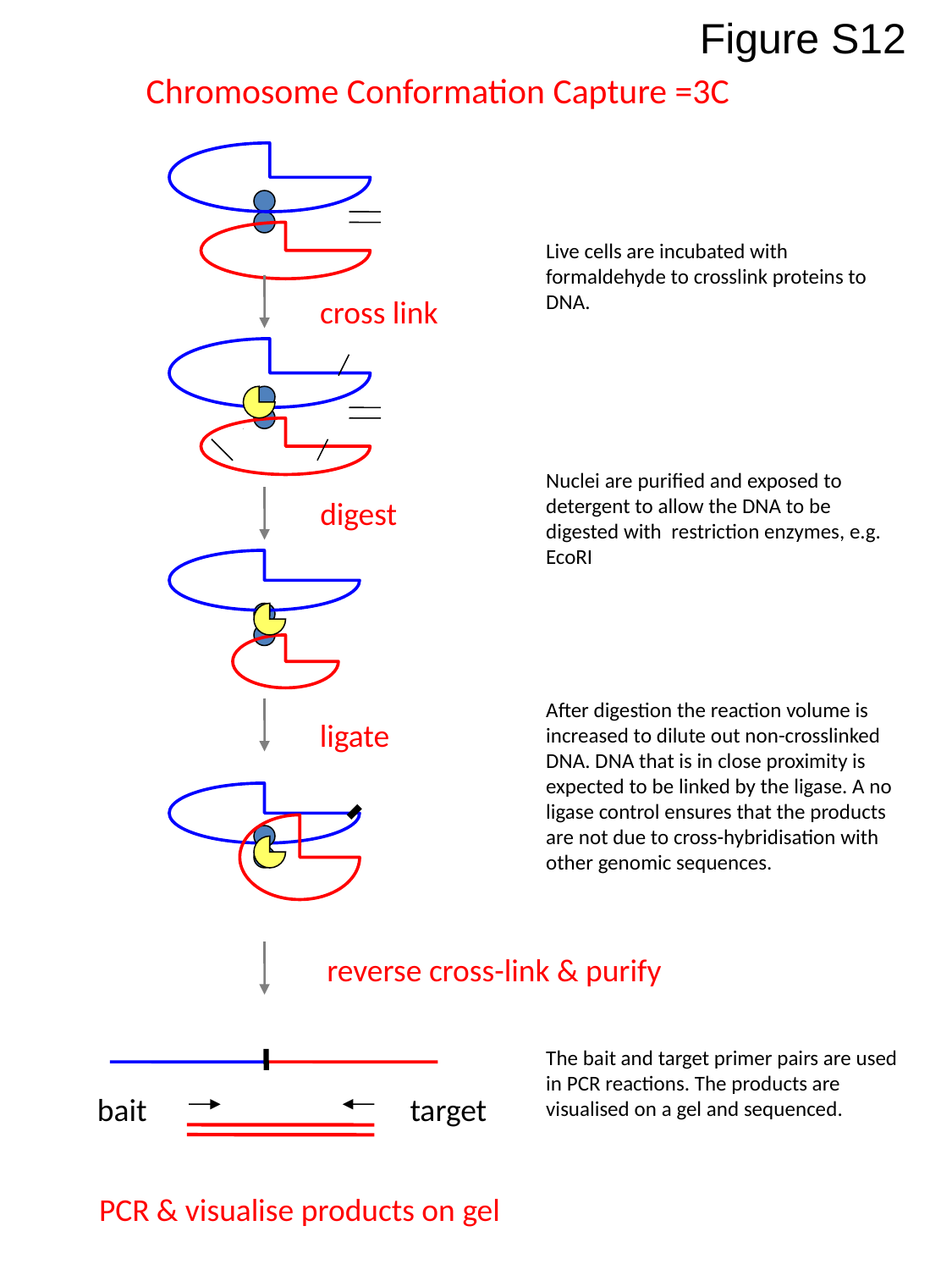

Figure S12
Chromosome Conformation Capture =3C
Live cells are incubated with formaldehyde to crosslink proteins to DNA.
cross link
Nuclei are purified and exposed to detergent to allow the DNA to be digested with restriction enzymes, e.g. EcoRI
digest
After digestion the reaction volume is increased to dilute out non-crosslinked DNA. DNA that is in close proximity is expected to be linked by the ligase. A no ligase control ensures that the products are not due to cross-hybridisation with other genomic sequences.
ligate
reverse cross-link & purify
The bait and target primer pairs are used in PCR reactions. The products are visualised on a gel and sequenced.
bait
target
PCR & visualise products on gel
