# Supplementary material for: A Functional Variant at a Prostate Cancer Predisposition Locus at 8q24 Is Associated with PVT1 Expression
Source: PLoS Genet. 2011 Jul 21;7(7):e1002165. doi: 10.1371/journal.pgen.1002165 (PMC3140991; doi:10.1371/journal.pgen.1002165)
Supplement: Table S1 — Oligonucleotides used in this study. (DOC) [file pgen.1002165.s013.doc]

Oligonucleotides used in this study

| 3C experiment |  |
| --- | --- |
| Bait  Targets:  MYC-DHS  MYC-P  PVT-P  FAM84B  -ve  Ligation Control | Ctgctcaaaaatgcctttgg  Gtttgtgtggcatgctgaag  Ccatggtccaaaatgaggtt  Attctgcagcagcacaactg  Gactgcaggagcaagaaagg  gcagtggaatggggaaagta  agacccactccaggcctact |
| Cloning |  |
| S-DHS cloning -forward  S-DHS cloning -reverse | (GCGGTCGAC)CGCAAGACAACAAACTGGTC  (GCGGGATCC)AGCCAGGTGCGGGATATAA |
| EMSA | (only the forward strand is given) |
| rs378854:  Minor allele  Common allele  YY1  OCT  C/EBPα  SP1 | GAAGTAAGAAAAATGTCGGGGAGGAGCCAA  GAAGTAAGAAAAATGCCGGGGAGGAGCCAA  gccgataagacgccattttaagtcctacgtca  GCCTTCGGGTATGCAAATTATTAAGTCT  GTTTGCAACAGCAGTAAAATGGGTCAAGGTT  ATTCGATCGGGGCGGGGGGAGC |
| ChIP: RT-PCR |  |
| GR-for  GR-rev  S-DHS for  S-DHS rev  S-DHS-ve for  S-DHs-ve rev | CcccctgCtCtgacatctt  cttttccgaggtggcgagtatc  gcacaggtcagtgggtgtg  cgagaagcgcaatattcagg  CTTCCTCCATTCAGGTCAGC  TGCAGTGGTTGTGGGAATAA |
| CNV analysis |  |
| PVT1-CNV-F  PVT1-CNV-R | ACCTGTAATCCCTTCCCAGTTT GCTCTGCATCCATATGAAGTTG |
